# Spatial and Feature-Based Attention in a Layered Cortical Microcircuit Model

**DOI:** 10.1371/journal.pone.0080788

**Published:** 2013-12-06

**Authors:** Nobuhiko Wagatsuma, Tobias C. Potjans, Markus Diesmann, Ko Sakai, Tomoki Fukai

**Affiliations:** 1 Zanvyl Krieger Mind/Brain Institute, and Department of Neuroscience, Johns Hopkins University, Baltimore, Maryland, United States of America; 2 Brain Science Institute, RIKEN, Wako, Saitama, Japan; 3 Institute of Neuroscience and Medicine, Computational and Systems Neuroscience (INM-6), Research Center Juelich, Juelich, Germany; 4 Brain and Neural Systems Team, RIKEN Computational Science Research Program, Wako, Saitama, Japan; 5 Faculty of Biology III, Albert-Ludwigs-University Freiburg, Freiburg, Germany; 6 Department of Computer Science, University of Tsukuba, Tsukuba, Ibaraki, Japan; 7 CREST, JST, Kawaguchi, Saitama, Japan; Instituto de Neurociencias de Alicante UMH-CSIC, Spain

## Abstract

Directing attention to the spatial location or the distinguishing feature of a visual object modulates neuronal responses in the visual cortex and the stimulus discriminability of subjects. However, the spatial and feature-based modes of attention differently influence visual processing by changing the tuning properties of neurons. Intriguingly, neurons' tuning curves are modulated similarly across different visual areas under both these modes of attention. Here, we explored the mechanism underlying the effects of these two modes of visual attention on the orientation selectivity of visual cortical neurons. To do this, we developed a layered microcircuit model. This model describes multiple orientation-specific microcircuits sharing their receptive fields and consisting of layers 2/3, 4, 5, and 6. These microcircuits represent a functional grouping of cortical neurons and mutually interact via lateral inhibition and excitatory connections between groups with similar selectivity. The individual microcircuits receive bottom-up visual stimuli and top-down attention in different layers. A crucial assumption of the model is that feature-based attention activates orientation-specific microcircuits for the relevant feature selectively, whereas spatial attention activates all microcircuits homogeneously, irrespective of their orientation selectivity. Consequently, our model simultaneously accounts for the multiplicative scaling of neuronal responses in spatial attention and the additive modulations of orientation tuning curves in feature-based attention, which have been observed widely in various visual cortical areas. Simulations of the model predict contrasting differences between excitatory and inhibitory neurons in the two modes of attentional modulations. Furthermore, the model replicates the modulation of the psychophysical discriminability of visual stimuli in the presence of external noise. Our layered model with a biologically suggested laminar structure describes the basic circuit mechanism underlying the attention-mode specific modulations of neuronal responses and visual perception.

## Introduction

Visual attention is a function of the brain that boosts our perception by selectively enhancing neuronal responses to particular visual stimuli [1]–[7]. It enables the brain to attend to the momentarily most important information [8], thereby enhancing perception in a number of aspects [1], [9]–[12]. Visual attention functions in two distinct modes: the spatial and the feature-based mode. Numerous studies have shown that directing attention to the spatial location or the distinguishing features of a visual target differentially enhances the related neural responses and the discriminability of visual stimuli [13]–[16]. For instance, neurons in visual cortices respond strongly to a bar presented in their receptive fields and aligned with their preferred orientation [3], [17]. If the subject attends to the spatial location of the bar, the gain of neuronal responses is boosted in an arbitrary orientation, including the preferred one. In contrast, if the subject attends to a specific feature of the visual stimulus (i.e., its orientation or direction of motion), the sharpness of the cells' tuning curve is enhanced, implying that the cells' response gain is increased around its preferred feature but is decreased around the orthogonal feature [13], [14]. Network models have been proposed to explain the attentional modulation of visual responses [3], [18]–[29]. However, the underlying mechanisms of the different attentional modes are poorly understood.

Previously, we constructed a network model with a pair of layered microcircuit models [30] to account for the classical experimental results on visual attention reported by Reynolds et al. [3]. Here, we extend the results of the previous models primarily in two aspects. First, we explore the mechanisms of different response modulations in the spatial and feature-based modes of attention. We hypothesize that such differences emerge from differential top-down influences on visual cortical networks, rather than from the presence of different neural circuits specialized for the two modes of attention. Second, we study the effects of attention for orientation selectivity by constructing networks of multi-layered cortical microcircuits of integrate-and-fire neurons with a biologically plausible cortical laminar structure [31]. We modeled each microcircuit (e.g., the relative cell populations and connection probabilities in its individual layers) based on the anatomical and electrophysiological properties of cortical microcircuits [32]–[34]. Some of these data were obtained from the rodent neocortex, which does not have a clearly distinguished columnar organization [35], [36]. Therefore, each microcircuit model describes a functional group of neurons with similar characteristic responses, but does not necessarily describe a spatially grouped neuron population such as a cortical column.

In the visual pathway the bottom-up input carrying sensory information projects to cortical layers 4 and 6 (L4 and L6), whereas the top-down input from higher visual areas carry attentional information to L2/3 and L5, avoiding L4 [28], [37]–[39]. Conversely, the output to higher cortical areas arises from the L2/3, L5, and L6 of the lower areas, and neurons in L2/3 mediate the synaptic interactions among functionally grouped microcircuits within the visual cortex. Since the top-down influence of attention has been suggested for neuronal responses in visual area V4 [40], response modulations in the spatial and feature-based modes of attention may arise from complex interactions between bottom-up sensory and top-down attentional inputs within layered cortical networks [14], [28]. Our cortical microcircuit model allows us to explore how these 2 inputs, which are distributed differently across functional microcircuits, interact mutually through inter-laminar and inter-microcircuit (inter-mc) synaptic connections. Due to a limitation of simulation resources, our model is restricted to a portion of the visual cortical space sharing a common receptive field. Therefore, our model cannot deal with competition induced by bottom-up attention and saliency map among spatially distributed stimuli [41], [42]. However, our model partly takes into account the importance of bottom-up mechanisms of attention because it integrates the effects of bottom-up visual input and top-down attention in the cortical laminar structure. We demonstrate that the resultant attentional modulations are consistent with those observed experimentally. Furthermore, our model accounts for the differential effects of the spatial and feature-based modes of attention on visual discriminability [16].

## Results

**Figure 1:**
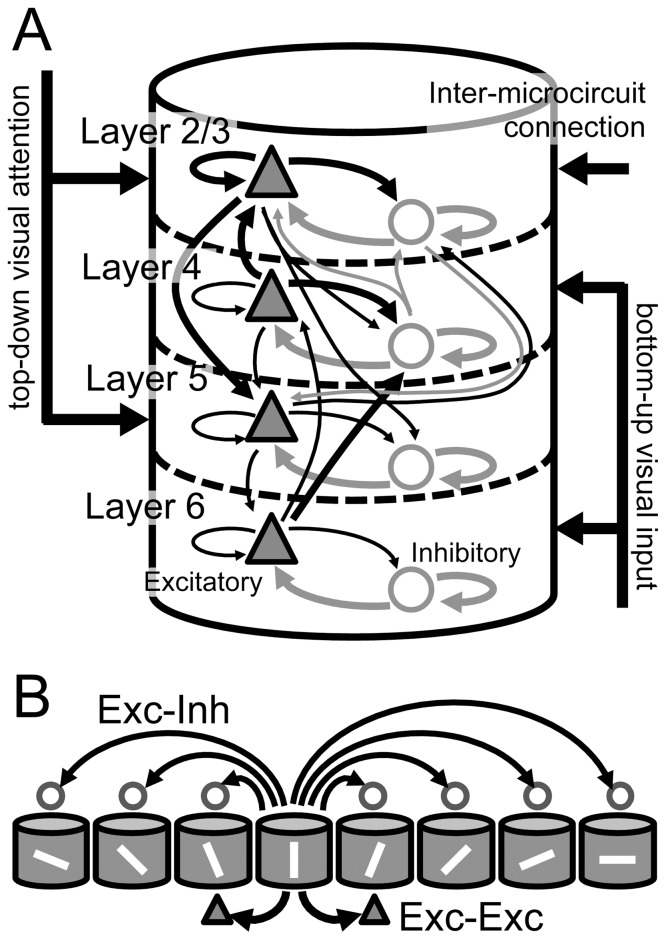
Model architecture of layered visual cortical microcircuits. ***A***, Intra- and inter-laminar synaptic connections and external inputs of a multi-layered microcircuit. Our model has 8 orientation-selective unit microcircuits, each of which comprises 20,000 integrate-and-fire neurons and constitutes L2/3, L4, L5, and L6. Triangles and circles represent excitatory or inhibitory neurons, respectively. Thick arrows represent strong synaptic connections with connection probability *C*>0.13, and narrow arrows represent synaptic connections with *C*>0.065. Other weaker synaptic connections are not shown. Layer 2/3 mediates inter-mc connections among orientation-selective microcircuits. Visual stimuli mimicking oriented bars project to both L4 and L6, while top-down attention projects to L2/3 and L5. ***B,*** Inter-mc synaptic connection of our model. Oriented bars on cylinders represent the preferred orientation of the individual unit microcircuits. The microcircuit model has two types of inter-mc connections mediated within L2/3: one is the lateral inhibition among microcircuits mediated by projections from excitatory neurons in 1 microcircuit to inhibitory neurons in the others (Exc-Inh). The other type is excitatory-to-excitatory connections between microcircuits with similar orientation selectivity (Exc-Exc). We had set a higher connection probability for Exc-Exc connections than for Exc-Inh connections.

**Figure 2:**
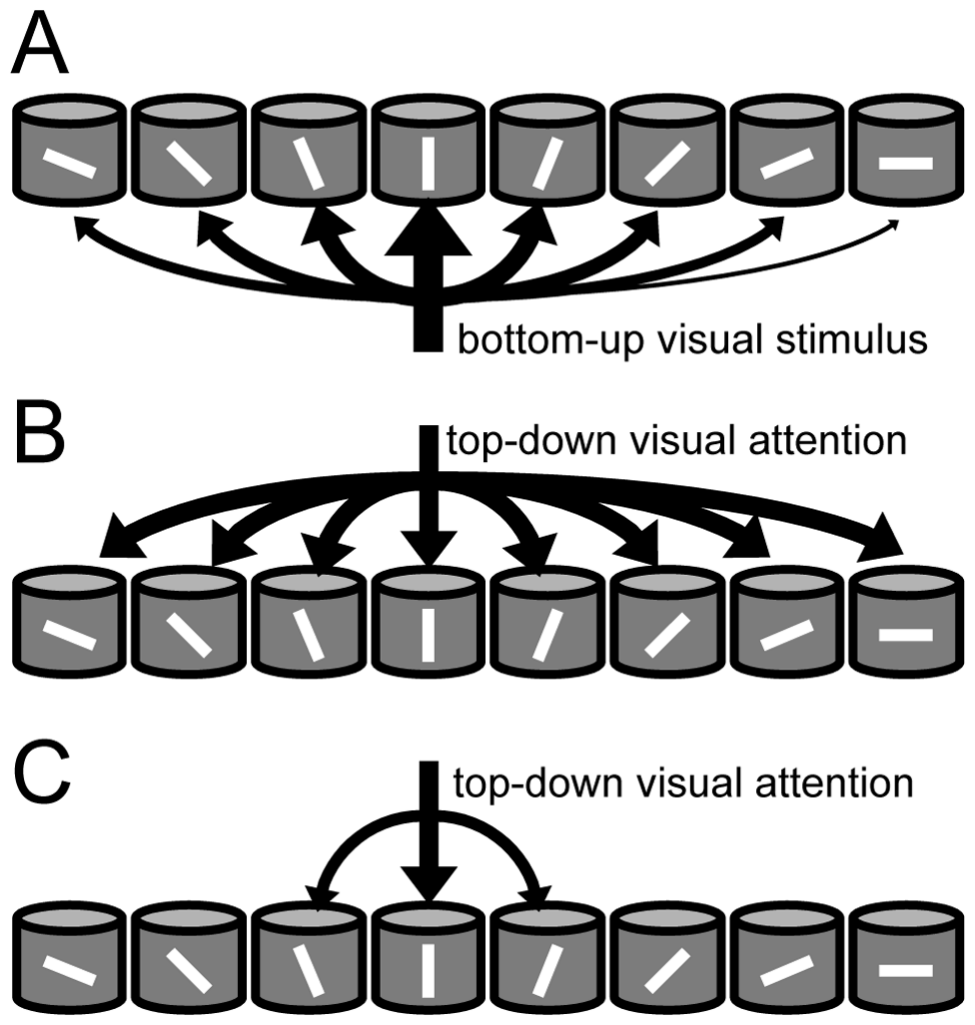
Visual stimuli and top-down input in our model. ***A*** Bottom-up visual inputs when a vertical bar is presented in the common receptive field of functionally grouped microcircuits are schematically illustrated. The thickness of each input represents its strength. The bottom-up inputs project most strongly to the microcircuits that prefer a vertical orientation and less strongly to the others. ***B,*** The top-down input mediating spatial attention is directed to the location of the model's receptive field and hence activates functionally grouped microcircuits. ***C,*** The top-down input mediating feature-based attention only projects to the microcircuits that prefer the attended orientation.

We previously constructed a model of the visual cortex by connecting 8 identical layered microcircuits (Figure 1) [30], [31]. Each layered microcircuit has L2/3, L4, L5, and L6, and each layer includes an excitatory pool and an inhibitory pool (Figure 1A). The 8 microcircuits have different preferred orientations, share the receptive field, and interact with one another through lateral inhibition and excitatory horizontal connections between microcircuits with similar orientation selectivity (Figure 1B) [35]. Our grouped microcircuit model is best suited for describing the neural networks of the primary visual cortex (V1) that are activated by oriented bars. A bottom-up sensory input representing an oriented bar projects to neurons in each microcircuit with different intensity depending on the stimulus orientation and the preferred orientation of the neurons (Figure 2A). Top-down input carrying spatial attention, i.e., attention to the location of the receptive field [13], is mediated by homogeneous projections to all microcircuits irrespective of their orientation selectivity (Figure 2B). In contrast, top-down input carrying feature-based attention, i.e., attention to an oriented bar in the location outside the cell's receptive field [14], projects selectively to the microcircuit that prefers the attended orientation (Figure 2C). Further details of the model are explained in the Materials and Methods section.

Further, we conducted some studies to understand the network mechanisms of attentional modulations in the neuronal responses observed in visual cortices [13], [14]. These previous studies involved complex visual stimuli that are difficult to replicate in a network model with only a single cortical area; the experimental results demonstrated similar effects of spatial and feature-based modes of attention on the tuning properties of neurons in different visual cortices. Therefore, in order to elucidate the underlying mechanism, we carried out simulations in a very simple case in which a variety of oriented bar stimuli were presented to subjects in this study. In each trial, the expected location (spatial attention) or the orientation of a stimulus (feature-based attention) was prompted by a visual cue for directing the animal's attention to the stimulus shown in the receptive field. Below, we explain the responses of model neurons in different scenarios.

### Neuronal responses of the microcircuit model under spatial attention

**Figure 3:**
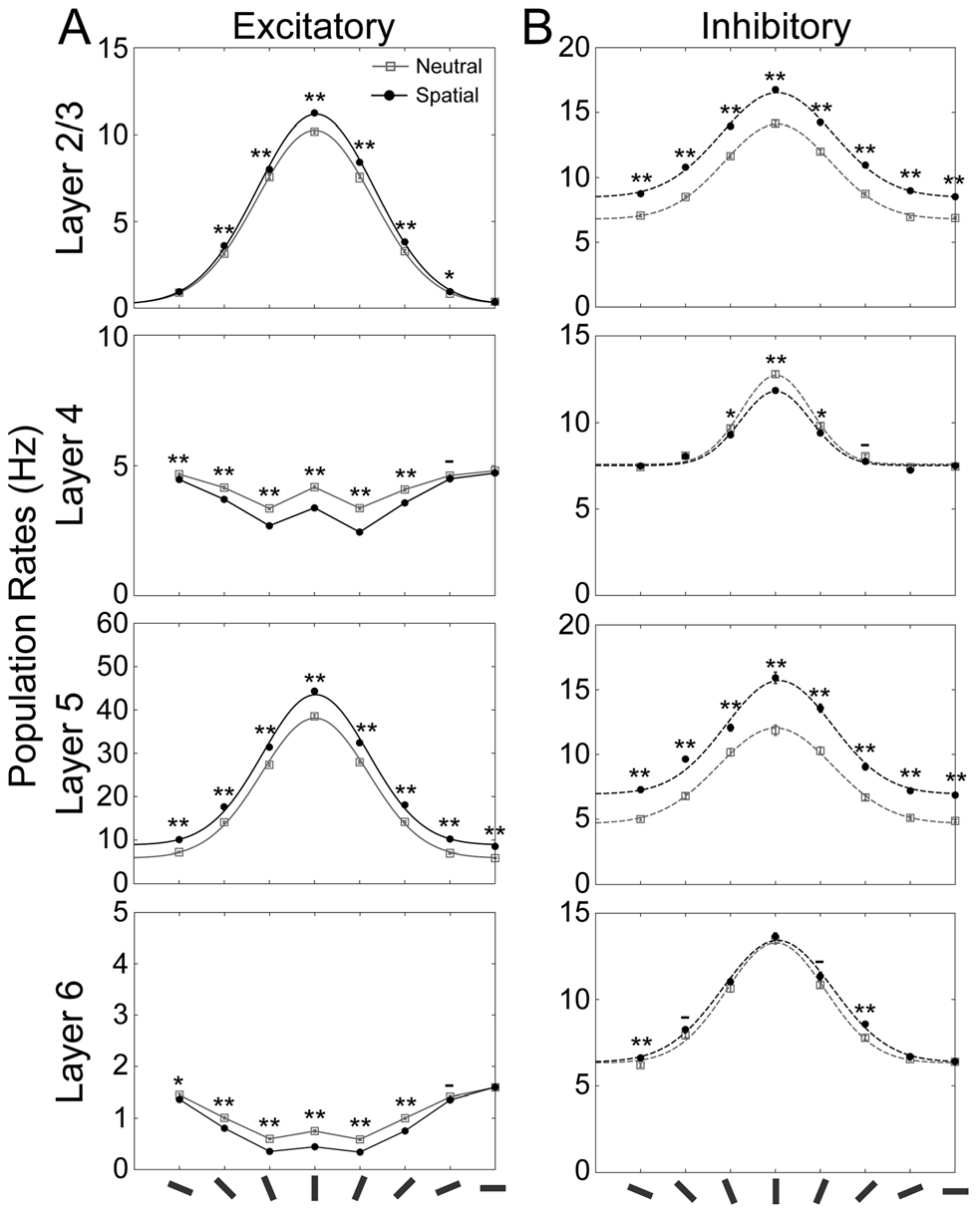
Model responses to a vertical bar for both neutral condition and spatial attention. The population rates of excitatory (***A***) and inhibitory (***B***) neurons for each layer of the microcircuit model are represented by solid and dashed lines, respectively. Oriented bars at the bottom present the preferred orientation of each layered microcircuit. Gray and black lines show the responses of the model without attentional input (neutral condition) or during spatial attention, respectively. The tuning curves of L2/3 and L5 excitatory neurons and inhibitory neurons in all layers were fitted to Gaussian distributions. Asterisks indicate that the differences in the population firing rates between the 2 conditions for this oriented bar was statistical significant (t-test: ** for p<0.01; * for p<0.05; – for p<0.1).

We first calculated the orientation tuning of neuronal responses under spatial attention. To this end, we applied a bottom-up sensory stimulus mimicking a vertical bar to L4 and L6 of microcircuits with preferred directions close to the vertical orientation. Top-down attentional input was applied with identical strength to L2/3 and L5 of all microcircuits (see Materials and Methods, Figure 1A and 2B). Figure 3 summarizes the mean population firing rates in the neutral condition (i.e., without attentional input) and under spatial attention (with attentional input) from 50 simulation trials. The average responses of neurons in microcircuits with different preferred orientations are plotted as a function of orientation. Because of the model's circular symmetry, the population tuning curve was equivalent to the tuning curve of single neurons responding to a bar stimulus presented in various orientations. For excitatory neurons in L2/3 and L5 and all inhibitory neurons, tuning curves are well approximated by Gaussian distributions.

**Figure 4:**
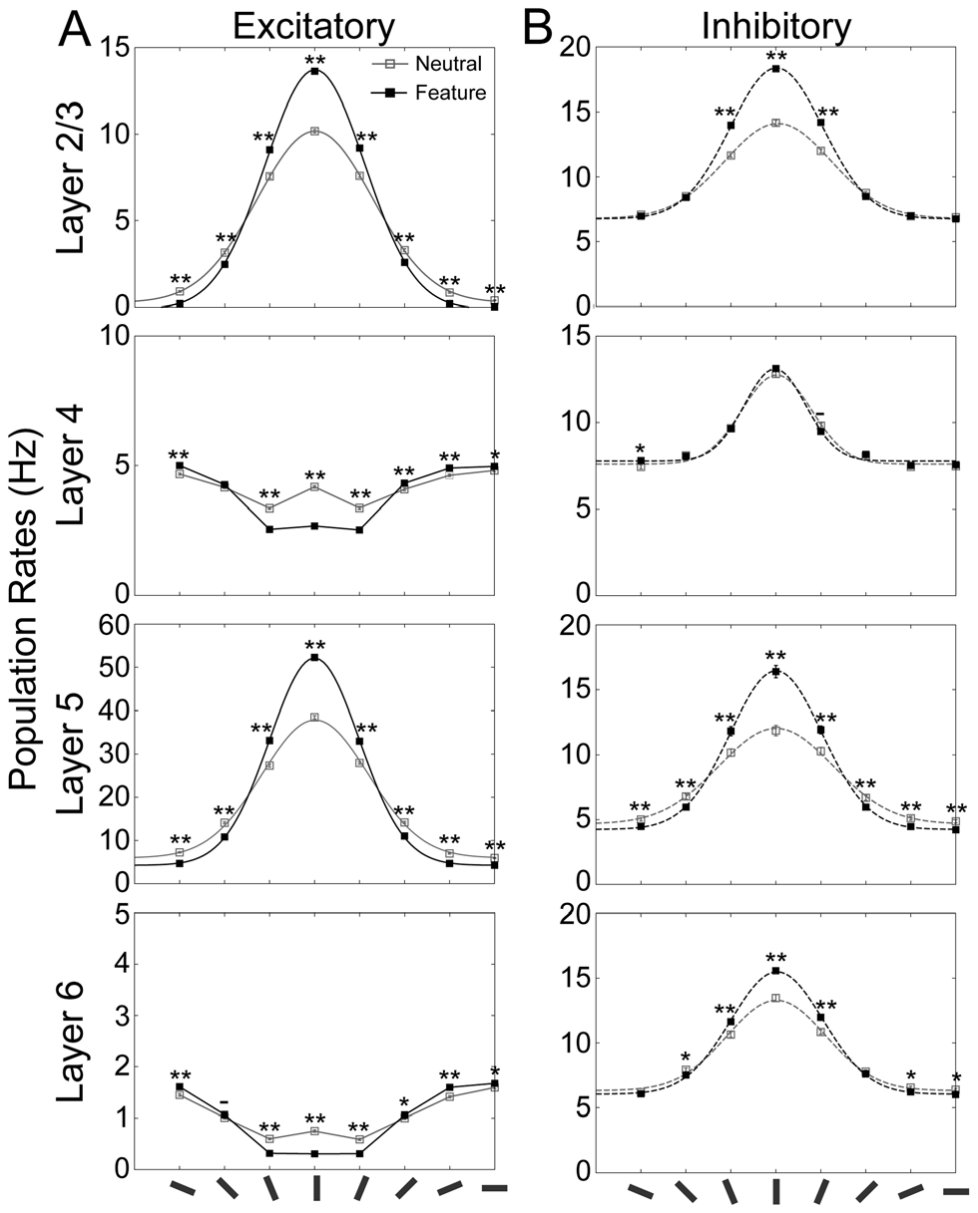
Model responses to a vertical bar for both neutral condition and feature-based attention. Population responses of excitatory (***A***) and inhibitory (***B***) neurons in the model. All the conventions are the same as those used in Figure 3. The model received a bottom-up input mimicking a vertical bar. The neuronal responses were compared between the 2 cases; i.e., in the neutral condition and in feature-based attention, where the responses in the neutral condition are identical to those shown in Figure 3. We used Gaussian distributions for curve fittings.

**Figure 5:**
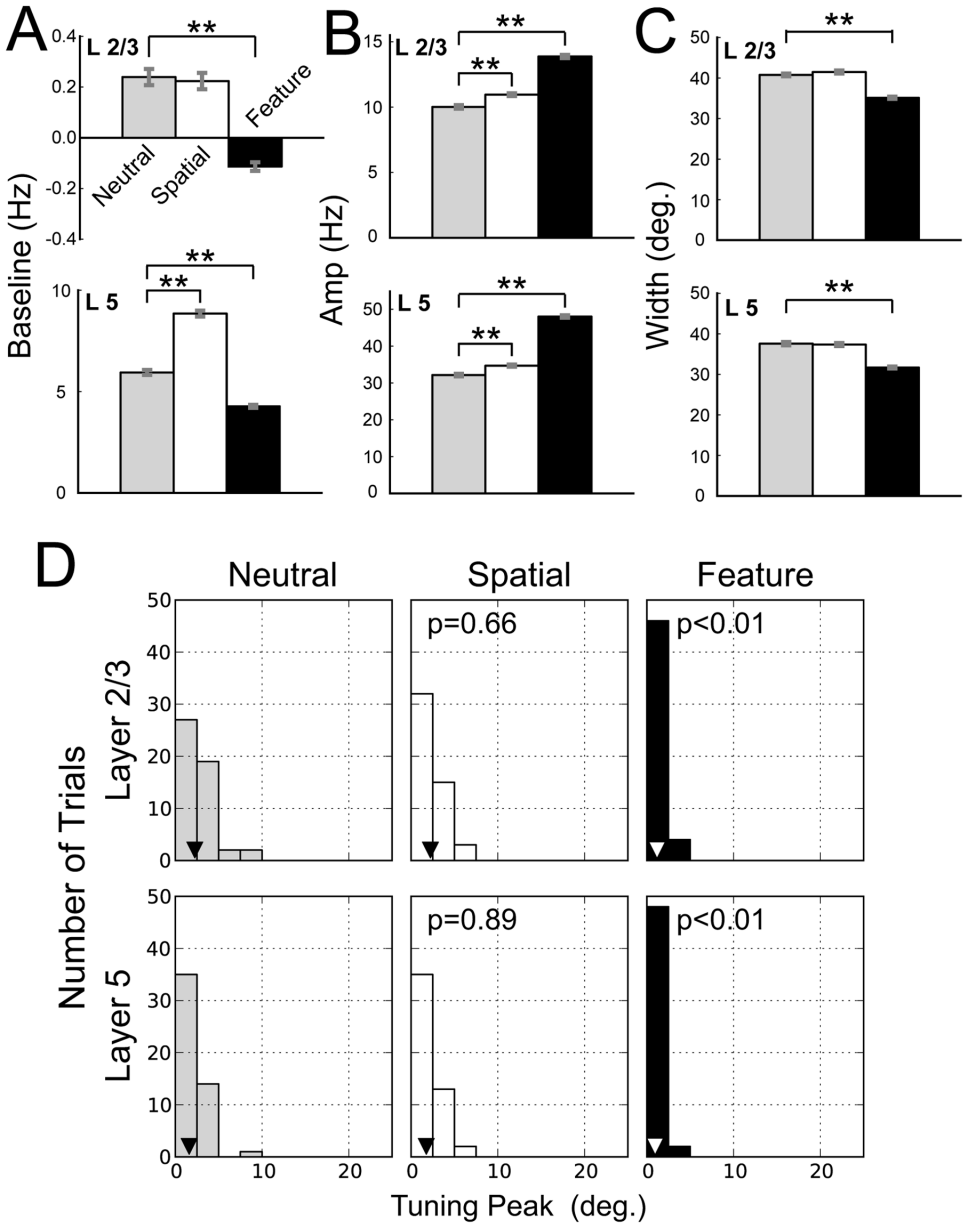
Statistical analyses of the orientation tuning curves. ***A, B, C,*** The baselines, amplitudes, and widths of the Gaussian tuning curves for the responses of excitatory neurons in L2/3 (upper) and L5 (lower) are shown. The values of the Gaussian fitting parameters were obtained from 50 simulation trials in the neutral condition (gray bars), spatial attention (empty bars), and feature-based attention (filled bars). Asterisks indicate that the parameter values were significantly different from those in the neutral condition (t-test: ** for p<0.01; * for p<0.05; – for p<0.1). ***D,*** The histograms of the absolute peak locations of the tuning curves in L2/3 and L5 are shown for a vertical bar stimulus. Triangles show the median values. We calculated *P* values for Mann-Whitney test to compare the histograms between the neutral condition and the 2 attentional conditions. Feature-based attention significantly improved the detection of the presented orientation.

**Figure 6:**
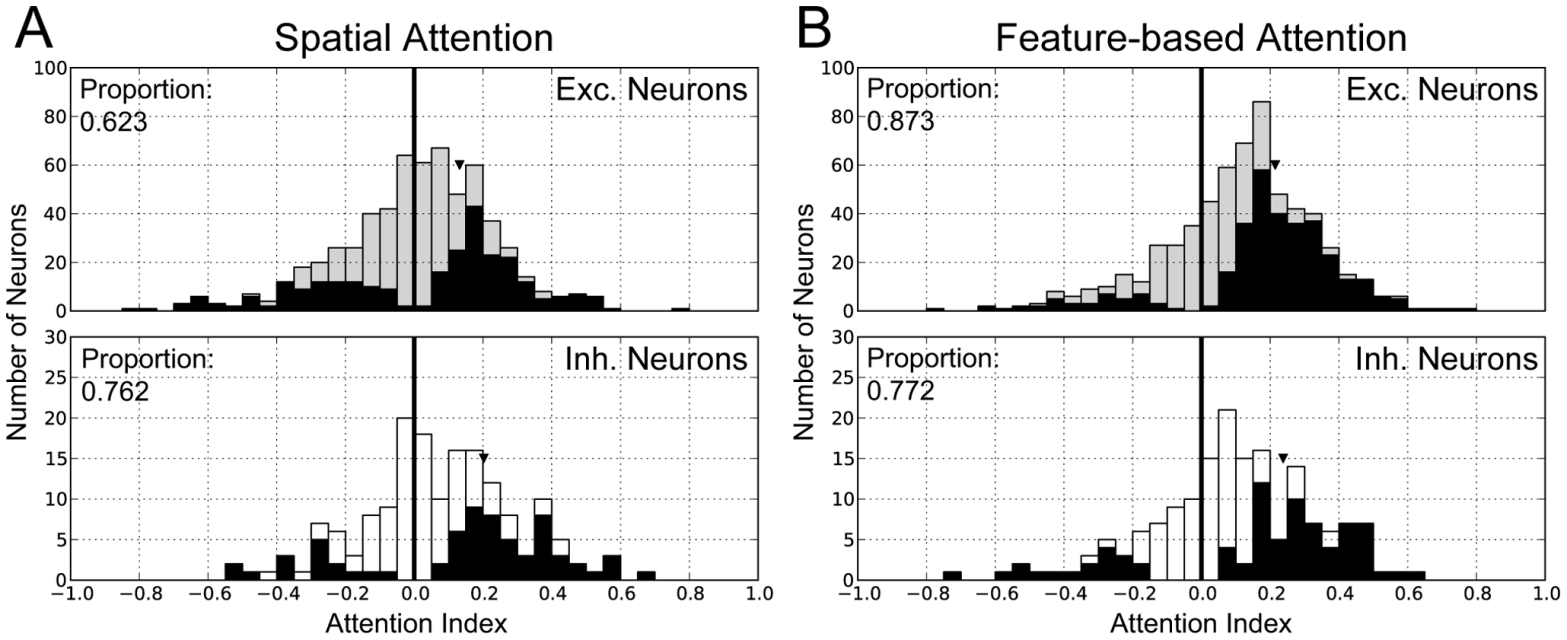
Distributions of attention indices (AIs) over excitatory and inhibitory neuronal populations in L2/3 and L5. ***A,*** The distributions of attention indices are displayed for spatial attention. The distributions of excitatory neurons (gray bars, upper panels) and those of inhibitory neurons (empty bars, lower panels) are depicted. In all panels, filled bars indicate the neurons showing statistically significant modulations by attention (t-test, p<0.01). Triangles show the median values. ***B,*** Similar distributions of attention indices are shown for feature-based attention.

**Figure 7:**
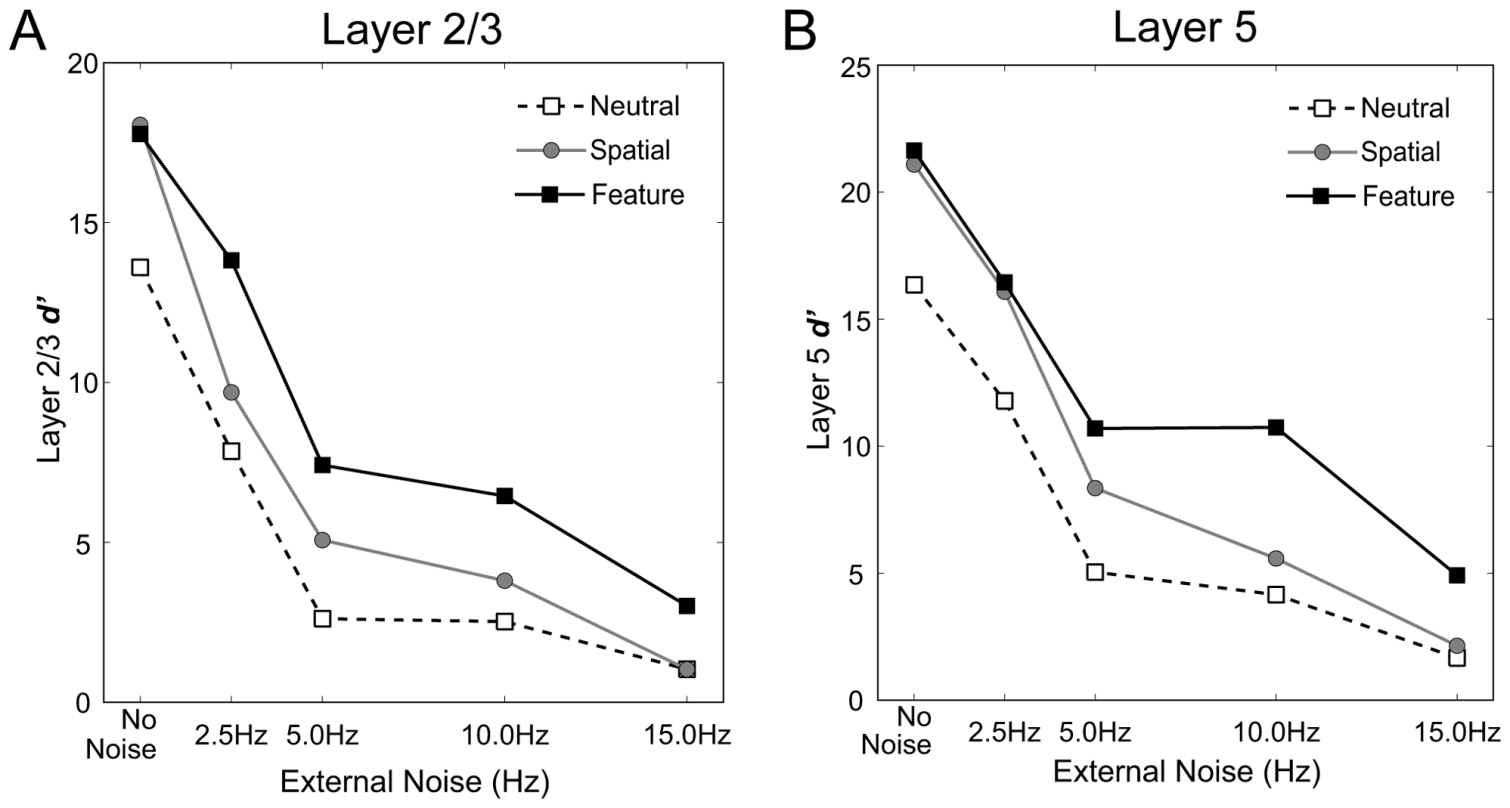
Influences of additional external noise on neuronal responses in our model. We carried out simulations at 4 different noise levels. We computed the discriminability index *d'* for L2/3 (***A***) and L5 (***B***) of the model responding to a vertical or a horizontal bar. The dashed line shows the magnitude of *d'* in the neutral condition. Solid gray and black lines indicate the results of spatial and feature-based modes of attention, respectively. L2/3 and L5 showed similar changes in discriminability with changes in the noise level. Only at a low noise level, spatial attention increased *d'* compared to *d'* in the neutral condition, whereas feature-based attention increased it at any noise level tested in this study.

Irrespective of orientation selectivity, spatial attention markedly enhanced the population activities in L2/3 and L5 of both excitatory and inhibitory neurons. This enhancement of the population firing rates in the model is consistent with results of several physiological experiments on spatial attention (cf. Figures 2, 4–7, and 10 in [13]). In the present model, excitatory neurons in L4 and L6 showed a contrasting difference to L2/3 and L5 neurons in the tuning curves as well as in their attentional modulations: top-down spatial attention significantly suppressed the population firing rates of L4 and L6. This suppression of L4, which was previously shown to be useful for a rapid shift of attention, occurs primarily due to an enhanced excitatory drive of L4 inhibitory neurons provided by L2/3 and L6 excitatory neurons [30]. Although our knowledge of the precise connectivity is limited, the connection probabilities of inter-laminar projections determined based on electrophysiological and anatomical data [32]–[34] predict that L4 and L6 of the visual cortex can exhibit different modulation patterns from those in other layers, possibly to accelerate the speed of analyzing visual objects with multiple complex features. This point is further discussed below.

### Neuronal responses of the microcircuit model under feature-based attention

Next, we calculated the orientation tuning of neuronal responses under feature-based attention. As in the simulations for spatial attention, a sensory stimulus mimicking a vertical bar was applied to the unit microcircuits with preferred directions close to the vertical orientation. However, attentional input was selectively applied to these microcircuits and not to others (see Materials and Methods and Figure 2C).

Figure 4 shows the population firing rates of different cortical layers under feature-based attention. The tuning curves shown for the neutral condition (gray lines) are identical to those in Figure 3. In L2/3 and L5, feature-based attention markedly enhanced the activity of those excitatory neurons in the microcircuits that received both the preferred visual stimulus and attentional input. In contrast, the activity of the excitatory cells in these layers that preferred horizontal orientations to vertical ones was significantly suppressed by attention. These results imply that feature-based attention increases both gain and selectivity of the tuning properties, which is consistent with the results of recent physiological experiments [14]. Again, we observed deviating tuning curves and modulation patterns for the excitatory neurons of L4 and L6.

### Statistical analyses of mode-dependent attentional modulations

For both spatial and feature-based modes of attention, the responses of excitatory neurons in L2/3 and L5 to oriented bars are well represented by Gaussian tuning curves. A Gaussian tuning curve has 4 parameters: the mean, standard deviation (SD), peak amplitude, and asymptote. The mean represents the preferred orientation, SD gives a measure of the tuning width, and the asymptote describes the baseline neuronal activity. In order to quantify the mode-dependent attentional modulations of our model, we statistically analyzed the 4 Gaussian parameters in different simulation conditions (i.e., neutral, spatial, and feature-based). To compare the results with those obtained in physiological experiments, we used the same statistical analysis method as used in ref. [13].

Figure 5 summarizes the results of the analysis for the neutral condition, spatial attention, and feature-based attention. The average magnitudes of the tuning baseline, amplitude, and width calculated from 50 simulation trials are displayed in Figure 5A, B, and C, respectively. Figure 5D presents the frequency histograms of the absolute value of the peak location (taken at the mean as an approximation) under the three simulation conditions. First, we compared the tuning properties of the neutral condition to those of spatial attention. For L2/3, the magnitude of the tuning amplitude was greater for spatial attention than for the neutral condition (t-test, p<0.01; Figure 5B). In contrast, there were no significant differences in tuning width (Figure 5C) and baseline (Figure 5A) between the neutral condition and spatial attention (t-test, p>0.1). These results demonstrate the marked effects of spatial attention on the gain of the population response in L2/3 combined with an invariance of orientation selectivity. For L5, we found a significant increase in tuning amplitude, as well as baseline (t-test, p<0.01), but no appreciable change in the tuning width (p>0.1). Therefore, it appears that spatial attention induces an overall enhancement of the amplitude of orientation tuning in L2/3 and L5. Since these layers are the output terminals of the visual cortical microcircuits, the enhancement of population firing rates will exert a strong impact on the activity of downstream visual cortices. We did not find any significant change in the peak location between the neutral and spatial attention (Figure 5D, Mann-Whitney test, p = 0.66 for L2/3 and 0.89 for L5). The overall enhancement of the tuning curve and the invariance of the tuning selectivity in spatial attention are in line with experimental results (see reference [13]; Figures 2B, 4, and 7).

As in spatial attention, feature-based attention significantly enhanced the amplitude of the tuning curves for both the layers (t-test, p<0.01; Figure 5B). In contrast to spatial attention, however, the magnitudes of both baseline (Figure 5A) and width (Figure 5C) of the tuning curves in L2/3 and L5 were smaller for feature-based attention than for the neutral condition (t-test, p<0.01). These results imply that selective top-down input to specific microcircuits not only boosts the gain but also sharpens the tuning curves of population responses in the output layers of the visual cortex. Such attentional modulations of gain and selectivity of the orientation tuning curves are commonly observed in electrophysiological experiments [14]. In our model, feature-based attention significantly shifted the peak locations in L2/3 and L5 towards the orientation of the stimulus (Figure 5D, Mann-Whitney test, p<0.01), indicating a significant improvement in the accuracy of analysis of stimulus orientation.

The response modulation patterns in L2/3 and L5 of our model showed good agreements with the biased competition observed in higher visual areas [3], [21]. Figure S1 summarized the mean firing rates of excitatory and inhibitory neurons in each layer of the microcircuit with a preference for vertical bars in various stimulus conditions for the biased competition. These responses of cortical layers are similar to those of our previous model [30]. Whether similar attentional modulations occur in V1, for which our model may best suit, remains unknown since small receptive fields of V1 neurons precluded such experiment [17].

### Role of the inter-mc connections in the two modes of attention

Our model could reproduce the qualitative and quantitative differences between spatial and feature-based modes of visual attention in terms of the orientation tuning curves. The distribution of top-down input over the 8 microcircuits was crucial for the mode-dependent attentional modulations. Further, we explored the role of inter-mc horizontal fibers in the two modes of visual attention by numerical simulations of a model that lacks inter-mc connections between L2/3 excitatory neurons. In these simulations, inter-mc lateral inhibition remained intact (Figure S2).

In spatial attention, the modulations of the orientation tuning curves in L2/3 and L5 were similar for the original as well as in the modified model (Figure S3). In addition, the tuning curves of these cortical layers exhibited a similar modulation under feature-based attention (Figure S4). In both the attention modes, excitatory neurons in L4 and L6 showed attentional modulation contrasting that shown by L2/3 and L5 neurons in the modified as well as the original model. Interestingly, in the modified model, the orientation tuning properties for L4 and L6 were preserved, unlike in the original model shown in Figure 3 and 4.

To obtain a quantitative insight into the behavior of the different models, we statistically compared the 4 parameters of the Gaussian tuning curves for L2/3 and L5 excitatory neurons in the neutral condition, spatial attention, and feature-based attention (Figure S5). In particular, spatial attention slightly, but statistically significantly, reduced the amplitude of the tuning curves for L2/3 in the modified model (Figure S5B, t-test, p<0.01). In addition, there was no significant difference in the width between the neutral condition and feature-based attention for both L2/3 and L5 in the modified model (Figure S5C, t-test, p>0.1). Because these modulation patterns seem to be inconsistent with the results of psychophysical experiments [16], we may conclude that the modified model is experimentally unacceptable.

Next, we carried out simulations of the modified model under various stimulus conditions for biased competition [3], [21]. Figure S6 showed the average firing rates of neurons in each layer of the microcircuit with vertical orientation selectivity. The modulation patterns for biased competition did not agree with physiological findings [3], [21]. In particular, the responses of excitatory neurons in L2/3 were not sensitive to visual stimuli and attention conditions. By contrast, a distractor horizontal bar enhanced the activities of L5 excitatory neurons, which implies that they were disinhibited (i.e., received a reduced inhibition) by a certain network mechanism. These results suggest that the inter-mc excitatory synaptic connections not only boost the population responses of unit microcircuits responding preferably to the presented stimulus, but also suppress the responses of microcircuits with the opposite preference.

### Response modulation of single neurons

We investigated the magnitudes of attentional modulation in individual excitatory and inhibitory neurons in L2/3 and L5 of a microcircuit with vertical preferred orientation. For the quantitative analyses and statistical tests, we used the normalized attention index (AI) defined as (A−U)/(A+U) for each neuron, where A and U are the firing rates of neuronal responses to attended and unattended stimuli [4], [5]. In each trial, the firing rates were averaged over the entire simulation period.

Figure 6A and 6B show the distributions of AIs for spatial attention and feature-based attention, respectively. Filled bars indicate the neurons that exhibit a statistically significant modulation in firing rate (t-test, p<0.05). All distributions were shifted toward the positive side by spatial, as well as feature-based attention. The median of AIs increased by 0.132 for excitatory neurons and by 0.202 for inhibitory neurons in spatial attention, while it increased by 0.215 for excitatory neurons and 0.238 for inhibitory neurons in feature-based attention. We note that, as in a previously proposed model [21], the modulation ratio defined as (1+AI)/(1−AI) is consistent in excitatory neurons with those obtained in electrophysiological recordings from MT [13].

Attentional modulation is also cell-type specific. In spatial attention, about 62% (169/271) of the significantly modulated excitatory neurons exhibited a significant attention-dependent increase in firing rate. About 76% (51/67) of the significantly modulated inhibitory neurons also showed such an increase. The difference in the proportion of positive and negative modulation between the two cell types is significant, according to a bootstrap test (1000 resamplings, p<0.001). Therefore, top-down spatial attention has a more consistent effect on the firing rates of inhibitory neurons than of excitatory neurons in L2/3 and L5. These differences in the attention-dependent response modulation between excitatory and inhibitory neurons are consistent with electrophysiological findings [5].

In the case of feature-based attention, about 87% (289/331) of the significantly modulated excitatory neurons showed a significant increase in firing rate, and about 77% (61/79) of the inhibitory neurons showed significantly increased firing rates. Again, a bootstrap test confirmed that this difference in the proportion of positive and negative modulation between excitatory and inhibitory neurons was statistically significant (1000 resamplings, p<0.001). Thus, top-down feature-based attention has a more consistent effect on the activity of excitatory neurons than of inhibitory neurons. As no experimental data are available, this constitutes a prediction of our model.

### Differential effects of the two modes of attention in visual perception

Biological systems are inevitably subjected to noise, and how a system responds to noisy input often reveals important characteristics of the system. Several studies have shown that additive external noise interferes with neuronal responses to noiseless visual stimuli and influences perception [16], [44]–[47]. Therefore, we explored how our microcircuit model responds to noisy visual stimuli. To this end, we added external noise that was uncorrelated with the orientation of the presented bar. A layered microcircuit with 0-degree preferred orientation received a bottom-up input mimicking a vertical bar. In addition to this input, other microcircuits received additional bottom-up inputs mediating external noise, which was given as a set of independent Poisson spike trains with a mean rate of 2.5, 5.0, 10.0, or 15.0 Hz.

Britten et al. could account for the psychophysical performance of monkeys by using neuronal responses in the visual area [44]. We assessed the performance of the model using the discriminability index (*d'*) for vertical and horizontal bars derived from the signal detection theory [48]. The index for the coarse discrimination represents the ability of the model to distinguish the 2 orthogonal orientations and is defined as
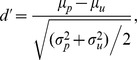
where 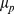 and 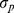 indicate the mean and SD of the population firing rates in the 0-degree preferential microcircuit responding to a vertical bar. Similarly, 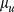 and 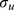 are the mean and SD of neuronal responses in the same microcircuit to a horizontal bar. We presented bottom-up visual signals mimicking either a vertical or a horizontal bar with various levels of external noise and investigated whether our model could discriminate the two oriented bars. Figure S7 presents an example of the histograms for population firing rates of the microcircuit that preferred a vertical bar, as shown in the analysis of the physiological experiment (cf. Figure 5 of [44]). We note that the responses of this microcircuit to a non-preferred stimulus (horizontal bar) can be inferred from the response to a vertical bar of the different microcircuit that prefers a horizontal bar [44]. The discriminability indices computed for neuronal responses in L2/3 and L5 are depicted in Figure 7A and 7B, respectively, as a function of the noise level in the neutral condition and the two modes of visual attention. Effects of attention on discriminability show a similar tendency in L2/3 and L5. In all cases, the discriminability between two orthogonal orientations decreases with an increasing level of external noise.

Compared with the neutral condition, under spatial attention, our model exhibited a marked increase in *d'* at low noise levels (Figure 7), without showing an improvement in discriminability at high noise levels. In contrast, under feature-based attention, the model showed a consistent enhancement of *d'* at both low and high noise levels. These results suggest that spatial attention improves perception of visual stimuli only at low levels of external noise, whereas feature-based attention improves it at both high and low noise levels. We also observed similar attention-mode dependent modulation by external noise in the distribution of the peak location (Figures S8 and S9). These differential effects of two kinds of attention are consistent with psychophysical observations [16].

To examine the performance of our model in more detail, we computed the values of *d'* for fine discrimination between vertical and 22.5-degree oriented bars as a function of the external noise level (Figure S10). Effects of the two modes of attention on the discriminability and the influences of the noise level are similar to those observed in the discrimination of orthogonal orientations shown in Figure 7. However, the magnitude of *d'* is markedly lower for similar orientations than for orthogonal orientations. This tendency shows a good agreement with the characteristics of human perception. Thus, our model can perform a fine discrimination although it is harder than a coarse discrimination.

## Discussion

To explore the circuit mechanism of attention for visual perception, we constructed a visual cortical network model consisting of 8 multi-layered functional microcircuits containing about 20,000 integrate-and-fire neurons in each microcircuit (about 160,000 neurons in total). Layers 4 and 6 of the individual microcircuits receive bottom-up preferred stimuli representing different oriented bars, and L2/3 and L5 receive a top-down input mediating spatial or feature-based attention. In addition to these inputs, the microcircuits interact with each other through the excitatory horizontal connections and lateral inhibition implemented within L2/3. This architecture produces inter-mc competition for simultaneous presentation of two orthogonal oriented bars (Figure S1, [3]). We have shown, by numerical simulation, that neuronal activities in the L2/3 and L5 of our model well account for the distinct response modulations in visual cortices induced by spatial and feature-based modes of attention [13], [14]. Especially, the discriminability of noisy visual stimuli in our model is consistent with experimental observations [16].

### Mechanisms of attention-mode–dependent modulation of neuronal responses

The essence of our model is the hypothesis that spatial and feature-based modes of attention are differently implemented by top-down input to cortical microcircuits (Figure 1B and 1C). The top-down input mediating feature-based attention projected preferentially to a specific microcircuit to enhance the model's responses to the attended feature. In contrast, spatial attention was mediated by a homogeneous top-down input projecting to all the functional microcircuits that share their spatial receptive fields. Consequently, in feature-based attention, top-down input enhanced the population responses in L2/3 and L5 of the microcircuits, which preferentially respond to the attended oriented stimulus, while suppressing the responses of microcircuits with other preferred orientations by inter-mc lateral inhibition (Figure 4). This suppression across microcircuits was effectively magnified by inter-mc excitatory connections (Figures S1 and S6). In spatial attention, a homogeneous top-down input compensated the suppressive effects of lateral inhibition and the response gain increased in all the local microcircuits. Recently, Cohen and Maunsell [49] reported that feature-based attention coordinates neuronal activation in V4 across hemispheres, whereas spatial attention acts on local neural populations. These results seem to support the different projection patterns of top-down input in the two attention modes observed for our model.

Excitatory connections between microcircuits with similar orientation preferences are necessary for shaping their tuning properties in different modes of visual attention. It was hard to quantitatively replicate the attention-type–specific modulations of neuronal responses in microcircuit models without these horizontal connections, although the qualitative modulation patterns of population rates are similar for experimental findings [13], [14]. However, such modified model replicated neither the modulation of the amplitude of the orientation tuning curves in spatial attention nor the modulation of the width in feature-based attention (Figure S5). In addition, these models did not produce competitive effects between orthogonal oriented bars presented simultaneously in the receptive field (Figure S6). These modulation patterns were inconsistent with the results of physiological experiments [3], [13], [14]. The modified model lacks excitatory connections between microcircuits with similar orientation preferences, so that the external inputs such as bottom-up visual inputs and top-down attention directly determine the responses of the microcircuit in the L2/3 excitatory neurons. In our simulations, the top-down input was much weaker than the preferred stimulus (see Materials and Methods). Therefore, when the homogeneous top-down input mediating spatial attention and the vertical bar were applied to the modified model, the responses of the microcircuit with a preference for vertical bar were mainly determined by the strength of the bottom-up visual input. In contrast, for the microcircuit with a preference for horizontal bar, the top-down spatial attentional input seemed to be stronger than the bottom-up input to this microcircuit (Figure 2A and 2B) and had great effects on the modulation of their responses in L2/3 excitatory neurons. Furthermore, the activation of these neurons might suppress the responses of the microcircuit with a preference for vertical bar through the lateral inhibition. Consequently, as shown in Figure S3A, the effects of spatial attention on L2/3 excitatory activity for the modified model were grater in microcircuits with horizontal-bar preference than those with vertical-bar preference. This indicated that, in the modified model, spatial attention markedly enhanced the magnitude of the baseline, whereas this mode of attention slightly increased the magnitude of (baseline + amplitude). The modified model lackeds excitatory connections between microcircuits with similar orientation preferences (Figure S2), so that the total strength of external inputs such as visual inputs and top-down attention might determine the responses of the L2/3 excitatory neurons. In our simulations, the top-down input was much weaker than the preferred stimulus (see Materials and Methods). Therefore, when the homogeneous top-down input mediating spatial attention and the vertical bar were applied to the modified model, the responses of the microcircuit with a preference for vertical bar were mainly determined by the visual input. In contrast, for the microcircuit with a preference for horizontal bar, the top-down attentional input was more dominant than the bottom-up input to this microcircuit (Figure 2A and 2B) and had profound effects on their responses of L2/3 excitatory neurons. Furthermore, the activation of these neurons might suppress the responses of the microcircuit with a preference for vertical bar through the lateral inhibition. Consequently, as shown in Figure S3A, the effects of spatial attention on L2/3 excitatory activity were grater in microcircuits with horizontal-bar preference than those with vertical-bar preference. This indicates that, in the modified model, spatial attention markedly enhanced the magnitude of the baseline, whereas this mode of attention slightly increased the magnitude of (baseline + amplitude).The possible explanations for these unacceptable modulation patterns offor the modified model were the convergence of excitatory signals in L2/3 inhibitory neurons via external inputs such as visual attention and lateral inhibition. The top-down signal mediating attention projected to not only excitatory but also inhibitory neurons in L2/3 and L5 (Materials and Methods). Furthermore, in the modified model, the effects of spatial attention on L2/3 excitatory activity were the greatest in microcircuits with horizontal-bar preference (Figure 3A), which might strengthen the responses of L2/3 inhibitory neurons in other microcircuits through inter-mc lateral inhibition (Figure 1B). If these excitatory projections to L2/3 inhibitory neurons in the microcircuit with vertical-bar preference were significantly effective, these neurons might prevent L2/3 excitatory neurons in the same microcircuit with vertical-bar preference from being enough activated.

The present model predicts that L2/3 and L5, to which top-down input projects directly, exhibit similar orientation tuning curves and similar attentional modulation patterns (suppression or enhancement). In contrast, the tuning curves and their modulation patterns are somewhat different in L4 and L6. Attentional modulations in these layers seem to depend on the strength of intra-mc synaptic connections and inter-laminar connections [30]. For instance, neurons in L4 integrate bottom-up sensory inputs and feedback excitatory signals from L2/3 that terminate on L4 inhibitory neurons (Figure 1A). Therefore, the balance between these opposing inputs determines the tuning property of L4. Our previous simulations suggested that the different pattern of attentional modulation in L4 is advantageous for a rapid shift of attention between visual objects [30]. Whether the different layers exhibit different orientation tuning and attentional modulations need to be examined by further experiments.

### Attention-induced response modulation across neuron types and layers

While visual attention increased the overall population responses for L2/3 and L5 in our model, the individual neurons showed a wide variety of modulations in firing rate (Figure 6). The divergent behavior of L2/3 neurons is of significant interest, because it can be examined by optical recordings in awake animals. First, despite the fact that the top-down input projected more densely to excitatory populations than to inhibitory ones in spatial attention (see Materials and Methods), this attention induced stronger enhancement in inhibitory cell activity (Figure 6A). In recent experiment [5], the strongest attentional modulation occurred among fast-spiking neurons that were putative inhibitory neurons. Our results seem to be consistent with these experimental findings because it is reasonable to regard inhibitory neurons in our model as the most frequent interneuron subtype, which is fast-spiking interneurons [50].

Why does attention induce different response modulations in different classes of neurons? The wiring pattern of inter-mc synaptic connections gives a possible explanation of such differences. Spatial attention increases the average firing rate of excitatory neurons in the L2/3 of all microcircuits irrespective of their preferred orientations. The enhanced activity of L2/3 excitatory neurons in a unit microcircuit is distributed to L2/3 inhibitory neurons in itself and other unit microcircuits through intra-laminar excitatory-to-inhibitory connections and inter-mc lateral inhibition (Figure 1A and B). Consequently, an inhibitory neuron in L2/3 receives convergent excitatory signals from all microcircuits in the model. In contrast, an excitatory neuron in L2/3 only receives excitatory input from the microcircuit it belongs to and its neighbors. This difference in the convergence pattern of excitatory signals presumably results in the stronger activation of inhibitory neurons and the weaker activation of excitatory neurons during spatial attention. The response modulation of the different neuron types has not been experimentally studied for feature-based attention. For future experimental tests, our model predicts the proportion of excitatory and inhibitory neurons that are significantly modulated by feature-based attention (Figure 6B).

Spatial attention does not exert noticeable effects on L2/3 excitatory neurons in microcircuits with horizontal preference (Figure 3A). Because of this, the effects of spatial attention look as if they were multiplicative rather than additive in L2/3, but not in L5. The differential effects on L2/3 and L5 may partly arise from the differential activation patterns of inhibitory neurons in the layers: the baseline activity of L2/3 inhibitory neurons is elevated by spatial attention almost uniformly in all unit microcircuits, whereas that of L5 inhibitory neurons is raised slightly stronger in a microcircuit receiving visual input (a vertical bar) and its neighbors. Therefore, in L5 excitatory effects of visual input may be compensated by the non-homogenous activation of inhibitory neurons, thus increasing the baseline activity uniformly across microcircuit. However, in L2/3 the enhanced inhibition is not compensated by visual input in microcircuits with horizontal preference, hence producing non-uniform elevation of the baseline level. We may interpret the layer-dependent effects of spatial attention as the multiplicative gain modulation of the whole tuning curve (baseline + stimulus-driven amplitude) suggested in other models [18], [21], [54], [55], because such a effect should be weak if the neutral activity is low, which is indeed the case for L2/3 excitatory neurons (neutral activity <1.0 Hz: see Figure 3). However, the laminar structure of our model is complicated and an explicit relationship between the two mechanisms remains to be further clarified.

### Limitations of our microcircuit model

Our model, with its simplified input structure, is best suited for describing those neural networks of the V1 that are activated by oriented bars. However, there is little electrophysiological evidence for the spatial and feature-based modes of attentional response modulations in V1; therefore, we adopted experimental data obtained for visual areas higher than V1, with more complex stimuli such as random dots motion stimuli for the middle temporal (MT) [16]. However, external noise is shown to interfere with the perception of attended directed stimuli in motion perception by the MT in similar manner as with orientation perception by early visual areas [46], [51]–[53]. Furthermore, Cohen and Maunsell [49] recently showed similar attentional modulations of firing rates in V4 neurons responding to oriented Gabor patches. These findings encouraged us to hypothesize that spatial and feature-based modes of attention share similar mechanisms across different cortical areas and different realizations of oriented stimuli.

Cohen and Maunsell [49] also reported that both modes of attention decrease spike correlations between neuron pairs. Attention is known to involve synchronized oscillations in visual cortical neurons [56]–[58], and recent modeling studies with somewhat more biologically detailed neurons suggest that attention involves the modulation of gamma-band oscillations in visual cortical neurons [22], [23], [28], [29], [59]. The dynamic properties of synchronization and oscillations depend significantly on the biological details of model neurons, particularly, fast-spiking interneurons [60]–[62]. Because our network model consists of relatively simple neuronal models, further studies are needed to clarify how spatial and feature-based modes of attention modulate spike correlations and, hence, visual information processing.

Our microcircuit model did not distinguish between simple and complex cells, because neurons were connected randomly, according to the connection probabilities listed in Table 1–4. The responses of simple cells generally depend on the spatial location of stimulus presentation within the receptive field, whereas those of complex cells do not significantly depend on the stimulus location [63]–[65]. For the sake of simplicity of simulation settings in large-scale network models, we did not model the detailed spatial location of stimulus presentation in this study. The network mechanism to generate complex cells from the responses of simple cells has not yet been fully clarified, and the implementation of such a mechanism remains open for future studies.

**Table 1:** Connection probabilities within and between layers (between excitatory neurons).

	From			
To	L2/3e	L4e	L5e	L6e
L2/3e	0.1960	0.1405	0.0534	0.0126
L4e	0.0127	0.0859	0.0111	0.0750
L5e	0.1684	0.0680	0.1255	0.0338
L6e	0.0258	0.0349	0.0947	0.0664

The entry in layers *i* (row) and *j* (column) represents the probability that a neuron in layer *j* receives synapses from a neuron in layer *i*.

e, Excitatory; i, Inhibitory.

**Table 2:** Connection probabilities within and between layers (from excitatory to inhibitory neurons).

	From			
To	L2/3e	L4e	L5e	L6e
L2/3i	0.1669	0.0601	0.1250	0.0070
L4i	0.1144	0.1809	0.0055	0.1750
L5i	0.0722	0.0346	0.0937	0.0142
L6i	0.0603	0.0056	0.0459	0.1089

The entry in layers *i* (row) and *j* (column) represents the probability that a neuron in layer *j* receives synapses from a neuron in layer *i*.

e, Excitatory; i, Inhibitory.

**Table 3:** Connection probabilities within and between layers (from inhibitory to excitatory neurons).

	From			
To	L2/3i	L4i	L5i	L6i
L2/3e	0.2570	0.1041	0.0	0.0
L4e	0.0098	0.2405	0.0005	0.0
L5e	0.1030	0.0094	0.6233	0.0
L6e	0.0109	0.0275	0.0326	0.3728

The entry in layers *i* (row) and *j* (column) represents the probability that a neuron in layer *j* receives synapses from a neuron in layer *i*.

e, Excitatory; i, Inhibitory.

**Table 4:** Connection probabilities within and between layers (between inhibitory neurons).

	From			
To	L2/3i	L4i	L5i	L6i
L2/3i	0.2270	0.0853	0.0	0.0
L4i	0.0048	0.2644	0.0	0.0
L5i	0.0445	0.0036	0.5288	0.0
L6i	0.0017	0.0008	0.0132	0.2389

The entry in layers *i* (row) and *j* (column) represents the probability that a neuron in layer *j* receives synapses from a neuron in layer *i*.

e, Excitatory; i, Inhibitory.

### Comparison with previous models of visual attention and gain modulation

Several models have been proposed to account for the differential effects of the two kinds of visual attention studied here. Deco and his colleagues [66], [67] proposed a model consisting of 3 visual areas: V1, Posterior Parietal (PP), and Inferotemporal (IT) areas. In their model, dorsal and ventral visual pathways mediate the different types of attention. Boynton [18] developed a normalization model of visual attention by using simple equations and reproduced results of various electrophysiological experiments on spatial and feature-based modes of attention. In the model, attention multiplicatively modulated sensory neurons' responses: spatial attention was described by multiplying the contrast gain on the normalization process whereas feature-based attention was represented by the multiplication of a feature-similarity gain factor after the normalization. Reynolds and Heeger [68] also proposed a normalization model for visual attention, in which a gain modulation occurs before normalization. These models describe the functions of visual attention at the macroscopic level without specifying the microscopic-level circuit structure. We studied the mechanisms of the mode-specific attentional modulations in cortical microcircuit models with a biologically suggested laminar structure. In our model, the gain factors mediated by top-down input and the normalization effects mediated by the inter-mc inhibitory effects arise concomitantly to produce the overall attentional modulations in spatial and feature-based attention modes. The inter-mc inhibitory connections in our model implement a function similar to a divisive normalization process in Reynold's model [68].

Buia and Tiesinga [19] constructed a simple circuit model representing two parallel visual pathways, each of which comprises 1 excitatory and 2 inhibitory neurons. They modeled feature-based attention by introducing top-down projections to specific types of inhibitory neurons and spatial attention by modulating the contrast gain of visual stimuli. Therefore, unlike in our model, in their model, spatial attention is mediated via bottom-up visual processing. Ardid et al., [21] developed a spiking neuron network model consisting of MT and working memory areas. Their model demonstrated the same types of attentional modulation as we modeled. In their model, attentional signal was restricted to the specific neuronal populations having a preference for the attended feature. Therefore, unlike our model, their model does not assume separate sources for spatial attention and feature-based attention. While many models describe network mechanisms of visual attention, single neurons can also perform the multiplicative gain control characteristic to spatial attention. For instance, a power-law input-output function of a neuron was shown to generate a multiplicative gain change [54], [55]. Whether attentional modulations and the characteristic gain change arise from a network-level mechanism or a single-neuron mechanism remains open for future studies.

Many models were also proposed for accounting for attentional effects on human perception observed in psychophysical experiments. Ling et al., [16] proposed that attention influences perceptual threshold by changing the gain or the tuning of population response in visual area MT. However, this model does not explain how the two kinds of attention were expressed in visual areas. Wagatsuma et al., [11], [69] described abstract models to explain how spatial and feature-based attention may change the visual perception for the object without modeling the underlying circuit mechanism. The present model explained the psychophysical effects of noise on human visual perception based on a microcircuit-level model of visual systems.

## Conclusions

We have constructed a cortical neural network model of layered microcircuits based on an integrated connectivity map derived from anatomical and electrophysiological data to account for the modulations of neuronal responses and the influences on visual perception observed for spatial and feature-based attention. Our model shows that the allocation of top-down input and inter-mc synaptic connections are critical for determining the effects of the two modes of visual attention in different cortical layers. Though our model certainly oversimplifies many features of cortical microcircuits, it allows us to investigate the electrophysiological effects of visual attention, including neuron-type specific (i.e., excitatory vs. inhibitory) attentional modulations, and their psychophysical implications in visual perception.

## Materials and Methods

### Microcircuit model of functionally grouped layered visual cortical microcircuits

Figure 1A displays the major neuronal and synaptic components of our multi-layered cortical microcircuit model. The model consists of 8 orientation-selective microcircuits, each representing the basic functional unit of the visual cortex, sharing their receptive fields and responding preferentially to 1 of the 8 stimuli (0, 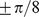, 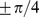, 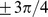, and 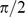 degrees). Each microcircuit has L2/3, L4, L5, and L6, and each layer consists of an excitatory neuron pool and an inhibitory neuron pool (see Table 5 for details). Layer 1 was not modeled explicitly, as it primarily contains the dendritic fibers of neurons in the other layers. Arrows in Figure 1A represent the major neuronal connections of this cortical microcircuit. Thick arrows show dense connections with a connection probability >0.13, while thin arrows represent connections with a connection probability <0.13 but >0.065. More sparse connections are not shown. The detailed connection probabilities are listed in Tables 1–4. For the sake of simplicity, all intra-mc connections have the same synaptic weight. The full network consisting of 8 functional microcircuits comprises, in total, about 160,000 integrate-and-fire model neurons, meaning that each microcircuit contains about 20,000 neurons. See Tables S1–S9 in File S1 for details of the neuronal models. The firing rate of model neurons would depend on the size of each unit microcircuit model since the total number of synaptic inputs to a neuron scales with the total number of neurons in the unit microcircuit [31]. In the present model, we adopted the architecture of our previous model [30], [31] after reducing the size of each unit microcircuit. Therefore, we rescaled the strength of all intra-mc synaptic connections by 1.6 times denser than the previous model in order to compensate for the reduction in the size of each microcircuit. The strength of external input was not modified. With these modifications, the spontaneous firing rate of each layer fell within a physiologically realistic range (L2/3: ∼3.3 Hz, L4: ∼2.4 Hz, L5: ∼15 Hz, L6: ∼0.6 Hz for pyramidal cells; L2/3: ∼8 Hz, L4: ∼6 Hz, L5: ∼9 Hz, L6: ∼8 Hz for inhibitory cells).

**Table 5:** Number of neurons in each layer of a single multi-layered microcircuit.

	Neuron types	
Layer	Excitatory neurons	Inhibitory neurons
L2/3	5171	1459
L4	5479	1370
L5	1213	266
L6	3599	737

This estimation of intra-mc synaptic connections was based primarily on anatomical and electrophysiological data for cat and rat cortices [32]–[34]. It is known that the rodent visual cortex does not have a columnar structure, which implies that neurons with similar orientation selectivity may not be spatially localized in the rodent brain [35], [36], [70]. However, the laminar structure, per se, is generally found in a variety of mammals, including the rat. If each layer of the visual cortex contains neurons playing similar functional roles across species, we may speculate that these neurons functionally have similar intra- and inter-laminar connectivity structures to form similar functional microcircuits. How we may obtain a consistent set of connection probabilities from the above data sets was demonstrated in Potjans and Diesmann [31] and Supplementary Materials.

Cortical L2/3 has rich local recurrent synaptic connections within the layer [71]. In addition, we introduced lateral inhibition among the L2/3 networks of the 8 layered microcircuits to induce competition among them [72]–[76]. As in a previous model [30], we introduced lateral inhibition by means of projections from L2/3 excitatory neurons of a microcircuit to L2/3 inhibitory neurons in others (Figure 1B), since, typically, only excitatory neurons make long-range connections, and cross-orientation suppression can be blocked by application of the GABA antagonist bicuculline [77]. The connection probability of the lateral connection was set to 0.03, irrespective of the orientation selectivity of the microcircuits. Excitatory horizontal connections are frequently found between columns or neurons showing similar preferred orientations [43], [76], [78]–[80]. Therefore, we introduced inter-mc excitatory connections between L2/3 excitatory neurons belonging to different microcircuits with similar orientation selectivity (Figure 1B). Due to the symmetric nature of interactions between neighboring microcircuits, the present model may be considered as a layered version of the so-called ring model [21], [22]. The strength (connection probability) of these synaptic connections was set to 0.06.

### Simulation experiments

When a visual stimulus mimicking an oriented bar is presented in the receptive field, excitatory and inhibitory neurons in L4 and L6 in the 8 layered microcircuits are excited with differential intensities (Figure 2A). The unit microcircuits were more strongly excited if their preferred orientations (0, 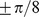, 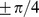, 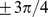, and 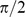 degrees) are closer to the orientation of the bar. Throughout this study, we regarded the orientation of a vertical bar as 0 and that of a horizontal bar as 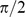. A preferred input was given as a set of independent Poisson spike trains of 20 Hz, and the firing rate was decreased for other stimuli, depending on the preference. Table 6 lists the probabilities that an L4 or an L6 neuron receives bottom-up sensory inputs. The population size of the bottom-up visual stimuli projecting to a microcircuit is about 225 fibers [30], [31].

**Table 6:** Projection probabilities of bottom-up sensory inputs.

	Sensory	
To	Excitatory neurons	Inhibitory neurons
L2/3	0.0	0.0
L4	0.0983	0.0619
L5	0.0	0.0
L6	0.0512	0.0196

Top-down excitatory signals mediating visual attention arrive at L2/3 and L5 of the microcircuits [30], [31], [39], [81]–[84] (Figure 1A). In our model, this top-down attentional pathway projects to both excitatory and inhibitory neurons. This makes the neuronal response of the present model multiplicative [85], [86], which explains the experimental observations. The top-down input was a set of low-frequency Poisson spike trains of 5 Hz. Thus, the top-down input was much weaker than the preferred stimulus [66], [67]. The number of spike trains in the top-down projection was 300 for both L2/3 and L5, and the connection probabilities of the top-down input are given in Table 7.

**Table 7:** Projection probabilities of top-down attentional inputs.

	Attention	
To	Excitatory neurons	Inhibitory neurons
L2/3	0.13	0.075
L4	0.0	0.0
L5	0.13	0.075
L6	0.0	0.0

In order to represent spatial and feature-based attention, we introduced 2 kinds of top-down input to our model. First, spatial attention was directed to a location within the receptive field of our model. In this case, we applied the same top-down input homogeneously to functionally grouped microcircuits, irrespective of their orientation selectivity (Figure 2B). In feature-based attention, the animal pays attention to 1 of the 8 orientations. Here, top-down input was delivered to the specific microcircuit that preferred the attended orientation (Figure 2C).

All simulation results were produced with the NEST Simulation Tool [87], using 8 cores (Intel Xeon^®^ 2.26 GHz) and MPI for parallel computation.

## Supporting Information

Figure S1 **The neuronal responses of the microcircuit model for the biased competitions [S13].** The population firing rates of excitatory (filled bars) and inhibitory (empty bars) neurons from 5 trials are shown for each layer of the vertical preferential microcircuit for various combinations of visual stimulus and feature-based attention. The preferred stimulus of the vertical preferential microcircuit is bordered white. An attended stimulus is circled. These modulation patterns in L2/3 and L5 are consistent with the experimental findings [S13]. Top-down attention signals induced in L4 a response modulation pattern opposite to that in L2/3 and L5. See our previous work [S2] for the detailed mechanisms and the analyses of the layer-dependence of the response modulations.
(TIF)

Figure S2 **The modified model without excitatory-excitatory connections among layered microcircuits.** In the case of this modified model, the interaction among microcircuits is mediated only by projections from L2/3 excitatory neurons in one microcircuit to L2/3 inhibitory neurons in the others (Exc-Inh).
(TIF)

Figure S3 **Responses of the modified model to a vertical bar for both neutral condition and spatial attention.** The population rates of excitatory ***A*** and inhibitory ***B*** neurons are presented in each layer of the modified microcircuit model by solid and dashed lines, respectively. Oriented bars at the bottom present the preferred orientation of each layered microcircuit. Gray and black lines show the responses of the modified model without attentional input (neutral condition) or during spatial attention, respectively. The tuning curves of excitatory and inhibitory neurons in all layers were fitted with Gaussian distributions. Asterisks indicate that the differences in the population firing rates between the two conditions is statistical significant (t-test: ** for p<0.01; * for p<0.05; – for p<0.1).
(TIF)

Figure S4 **The population responses of excitatory** ***A*** **and inhibitory** ***B*** **neurons in the modified model for both neutral condition and feature-based attention.** All the conventions are the same as those used in Figure S2. The modified model received a bottom-up input mimicking a vertical bar. The neuronal responses were compared between the two cases, i.e., in the neutral condition and in feature-based attention, where the responses in the neutral condition are identical to those sown in Figure S2. We used Gaussian distributions for curve fittings.
(TIF)

Figure S5 **Statistical analyses of the orientation tuning curves for the modified model.** ***A, B, C,*** The baselines, amplitudes and widths of the Gaussian tuning curves are shown for the responses of excitatory neurons in L2/3 (upper) and L5 (lower). The values of the Gaussian fitting parameters were obtained from 50 simulation trials in the neutral condition (gray bars), spatial attention (empty bars) and feature-based attention (filled bars). Asterisks indicate that the parameter values are significantly different from those in the neutral condition (t-test: ** for p<0.01; * for p<0.05; – for p<0.1). ***D,*** The histograms of the peak locations of the tuning curves in L2/3 and L5 are shown for a vertical bar stimulus. Triangles are the medians. We calculated *P* values for Mann-Whitney test to compare the histograms between the neutral condition and the two attentional conditions.
(TIF)

Figure S6 **The averaged neuronal responses of the modified model for the biased competitions from 5 simulation trials.** All the conventions are the same as those used in Figure S1. The modulation patterns in L2/3 and L5 are inconsistent with the results of physiological experiments [S13].
(TIF)

Figure S7 **Frequency histograms of population firing rates on the neutral condition obtained from L2/3** ***A*** **and L5** ***B*** **at five different levels of external noise.** The horizontal axis shows the amplitude of the population rates, and vertical axis indicates the number of trials. Each plot shows the levels of external noise. White bars depict population responses obtained from the preferred orientation, while black bars illustrate population responses to the non-preferred orientation. Each distribution is obtained from 50 simulation trials in the neutral condition. With increasing level of external noise, these two distributions were merged, which suggested the interferences of the accurate detection of a presented stimulus under the high level of noise.
(TIF)

Figure S8 **The histogram of the peak location of the tuning curves in L2/3 for the responses to the vertical bar with a variety of levels of external noise.** Triangles are the medians. We calculated *P* values for Mann-Whitney test to compare the histograms between the neutral condition and the two attentional conditions. ***A*** *,* The frequency histogram of tuning peaks of neutral condition. With increasing levels of external noise, the frequency is widely distributed. ***B*** *,* The histogram of the peak location of spatial attention with respect to the vertical bar with various levels of external noise. ***C*** *,* The histogram of the peak location of feature-based attention. Regardless of the level of external noise, feature-based attention improves the detection of the presented orientation compared to neutral condition.
(TIF)

Figure S9 **The histograms of the peak locations of the tuning curves in L5 obtained from same data sets shown in Figure S6.** All the conventions are the same as those used in Figure S6. The results of statistical test (Mann-Whitney test) to compare between the neutral condition and two kinds of visual attention are identical to Figure S6. ***A,*** The frequency histogram of tuning peaks of neutral condition. ***B,*** The distribution of the peak locations for spatial attention. ***C,*** The histogram of the peak locations of feature-based attention.
(TIF)

Figure S10 **The performance of our model using the discriminability index (** ***d'*** **) for vertical and 22.5-degree oriented bars (fine discriminability).** We computed the *d'* for L2/3 (***A***) and L5 (***B***). The dashed line shows the magnitude of *d'* in the neutral condition. Solid gray and black lines indicate the results of spatial and feature-based modes of attention, respectively. Effects of attention on discriminability show a similar tendency in L2/3 and L5. The decrement of this discriminability with increasing the levels of external noise was similar to the patterns of the discriminability between 2 orthogonal orientations. However, these magnitudes of *d'* between 2 similar oriented bars were markedly lower than that between orthogonal bars shown in Figure 7.
(TIF)

File S1 **This file contains Table S1-Table S9. Table S1,** Model description after [S4] (Model Summary). **Table S2,** Model description after [S4] (Population). **Table S3,** Model description after [S4] (Connectivity). **Table S4,** Model description after [S4] (Neuron and synapse model). **Table S5,** Model description after [S4] (Input). **Table S6,** Model description after [S4] (Measurements). **Table S7,** Spike rates of excitatory background inputs. **Table S8,** Neuronal and synaptic model parameters (Connectivity). **Table S9,** Neuronal and synaptic model parameters (Neuron and Synaptic model).
(DOCX)

Text S1 **Supporting Materials and Methods.**
(DOCX)
