# Supplementary figures and images for: Spatial and Feature-Based Attention in a Layered Cortical Microcircuit Model
Source: PLoS One. 2013 Dec 6;8(12):e80788. doi: 10.1371/journal.pone.0080788 (PMC3855641; doi:10.1371/journal.pone.0080788)

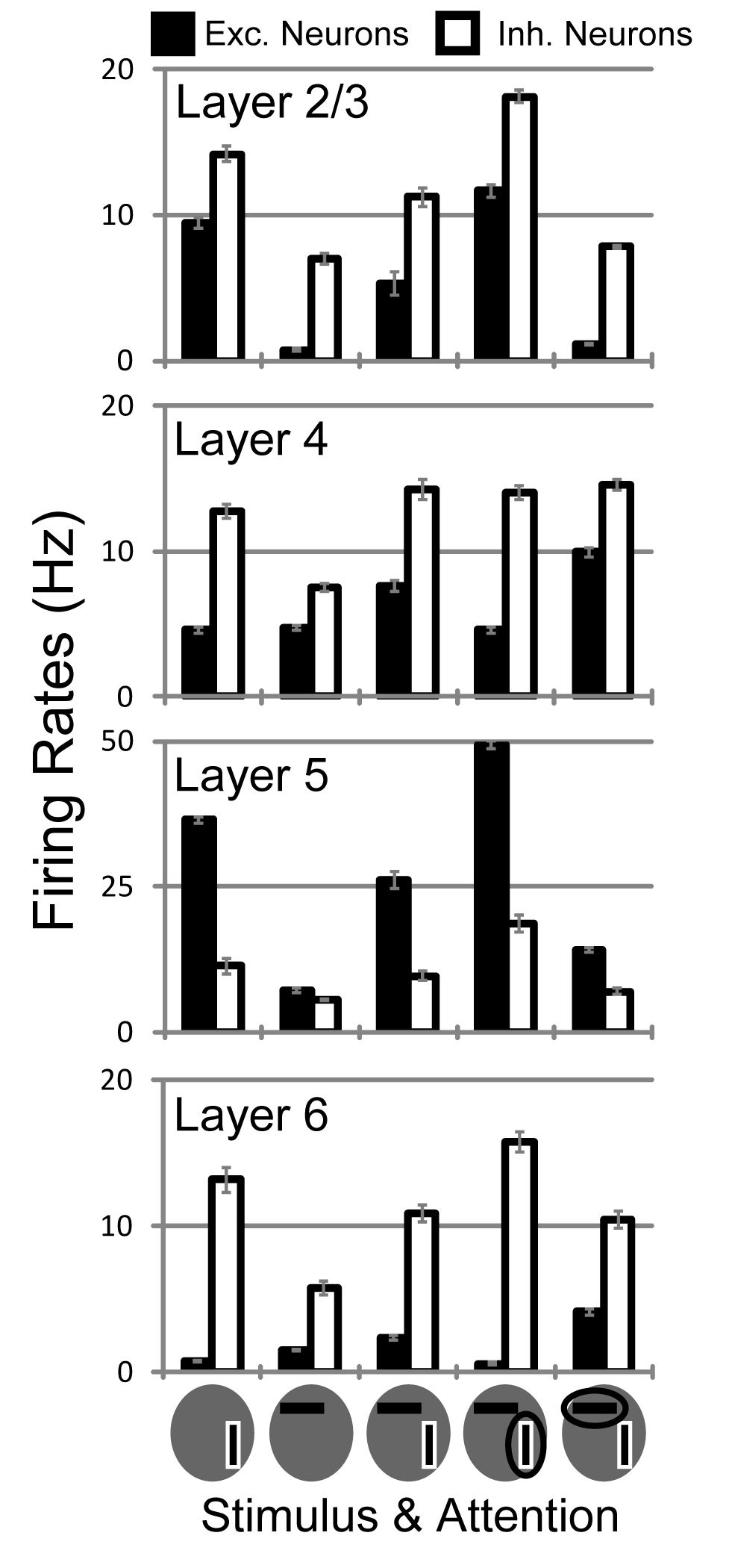

Supplement: Figure S1 — The neuronal responses of the microcircuit model for the biased competitions [S13]. The population firing rates of excitatory (filled bars) and inhibitory (empty bars) neurons from 5 trials are shown for each layer of the vertical preferential microcircuit for various combinations of visual stimulus and feature-based attention. The preferred stimulus of the vertical preferential microcircuit is bordered white. An attended stimulus is circled. These modulation patterns in L2/3 and L5 are consistent with the experimental findings [S13]. Top-down attention signals induced in L4 a response modulation pattern opposite to that in L2/3 and L5. See our previous work [S2] for the detailed mechanisms and the analyses of the layer-dependence of the response modulations. (TIF) [file pone.0080788.s001.tif]

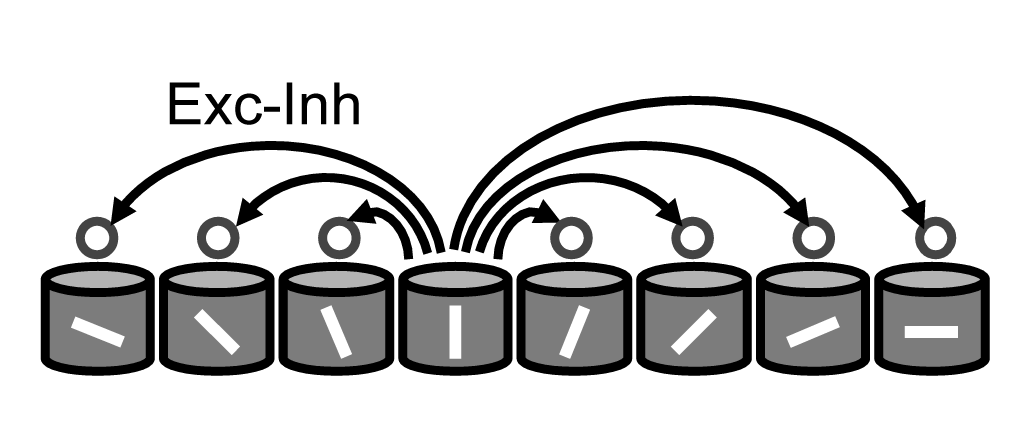

Supplement: Figure S2 — The modified model without excitatory-excitatory connections among layered microcircuits. In the case of this modified model, the interaction among microcircuits is mediated only by projections from L2/3 excitatory neurons in one microcircuit to L2/3 inhibitory neurons in the others (Exc-Inh). (TIF) [file pone.0080788.s002.tif]

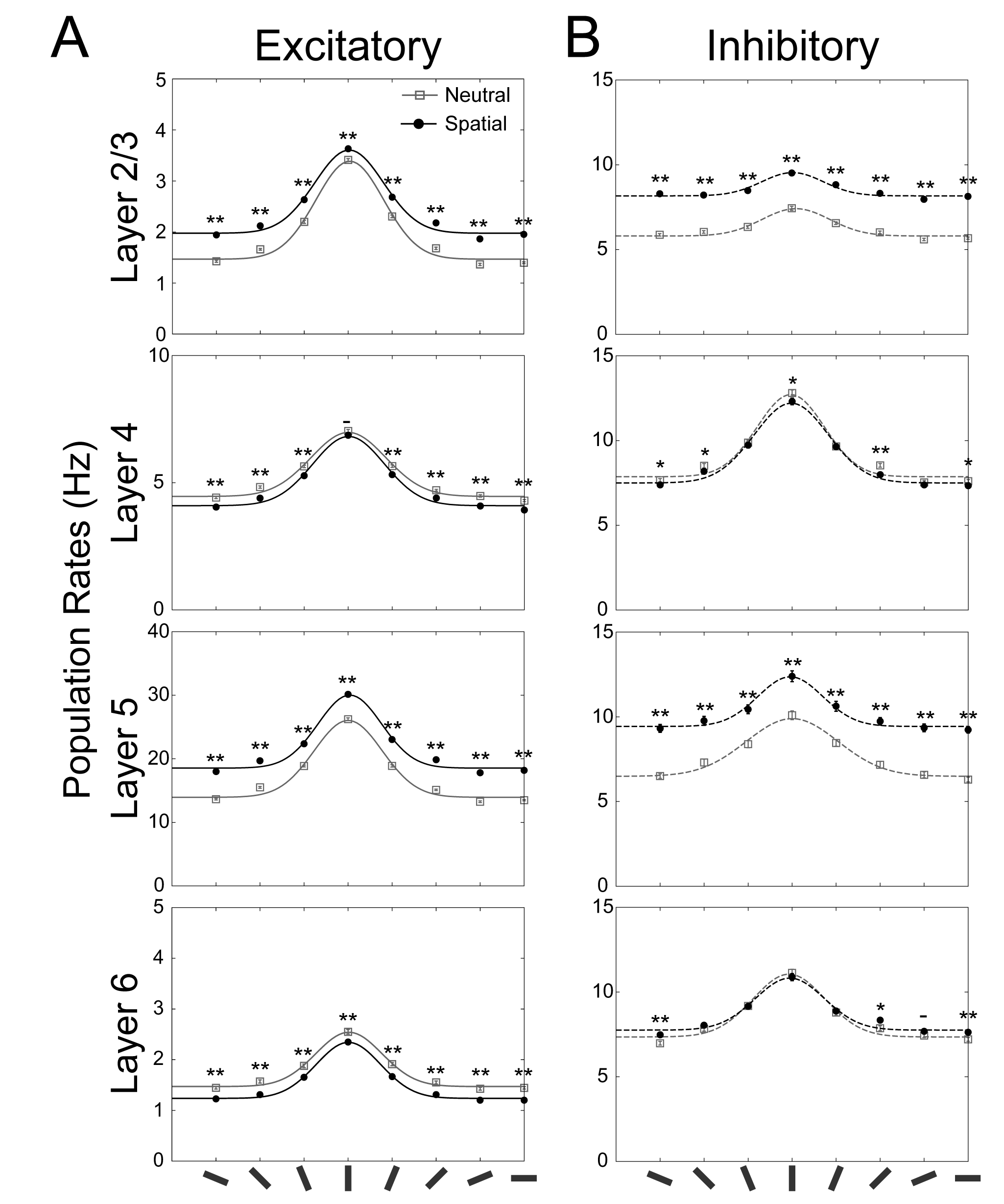

Supplement: Figure S3 — Responses of the modified model to a vertical bar for both neutral condition and spatial attention. The population rates of excitatory A and inhibitory B neurons are presented in each layer of the modified microcircuit model by solid and dashed lines, respectively. Oriented bars at the bottom present the preferred orientation of each layered microcircuit. Gray and black lines show the responses of the modified model without attentional input (neutral condition) or during spatial attention, respectively. The tuning curves of excitatory and inhibitory neurons in all layers were fitted with Gaussian distributions. Asterisks indicate that the differences in the population firing rates between the two conditions is statistical significant (t-test: ** for p<0.01; * for p<0.05; – for p<0.1). (TIF) [file pone.0080788.s003.tif]

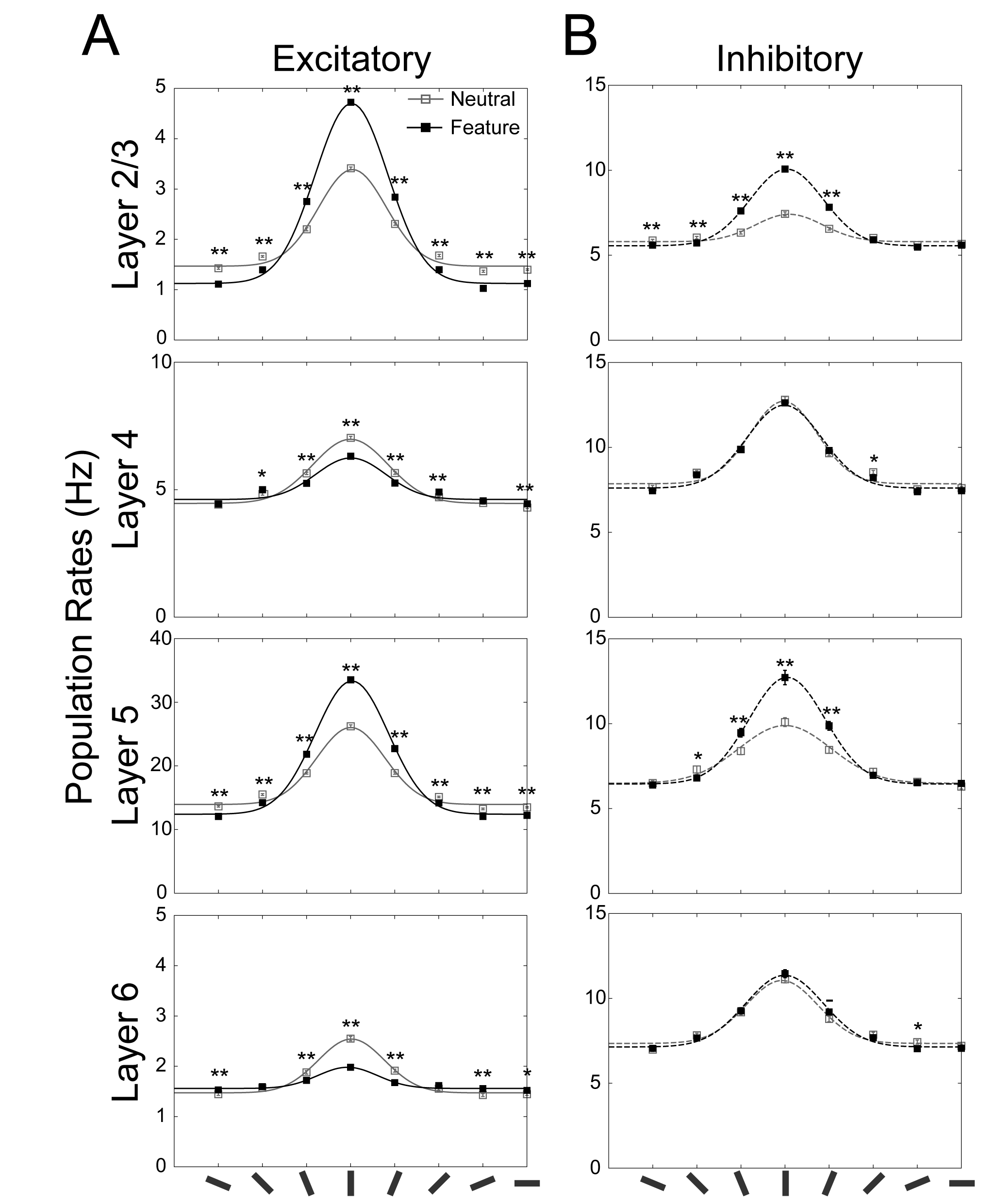

Supplement: Figure S4 — The population responses of excitatory A and inhibitory B neurons in the modified model for both neutral condition and feature-based attention. All the conventions are the same as those used in Figure S2. The modified model received a bottom-up input mimicking a vertical bar. The neuronal responses were compared between the two cases, i.e., in the neutral condition and in feature-based attention, where the responses in the neutral condition are identical to those sown in Figure S2. We used Gaussian distributions for curve fittings. (TIF) [file pone.0080788.s004.tif]

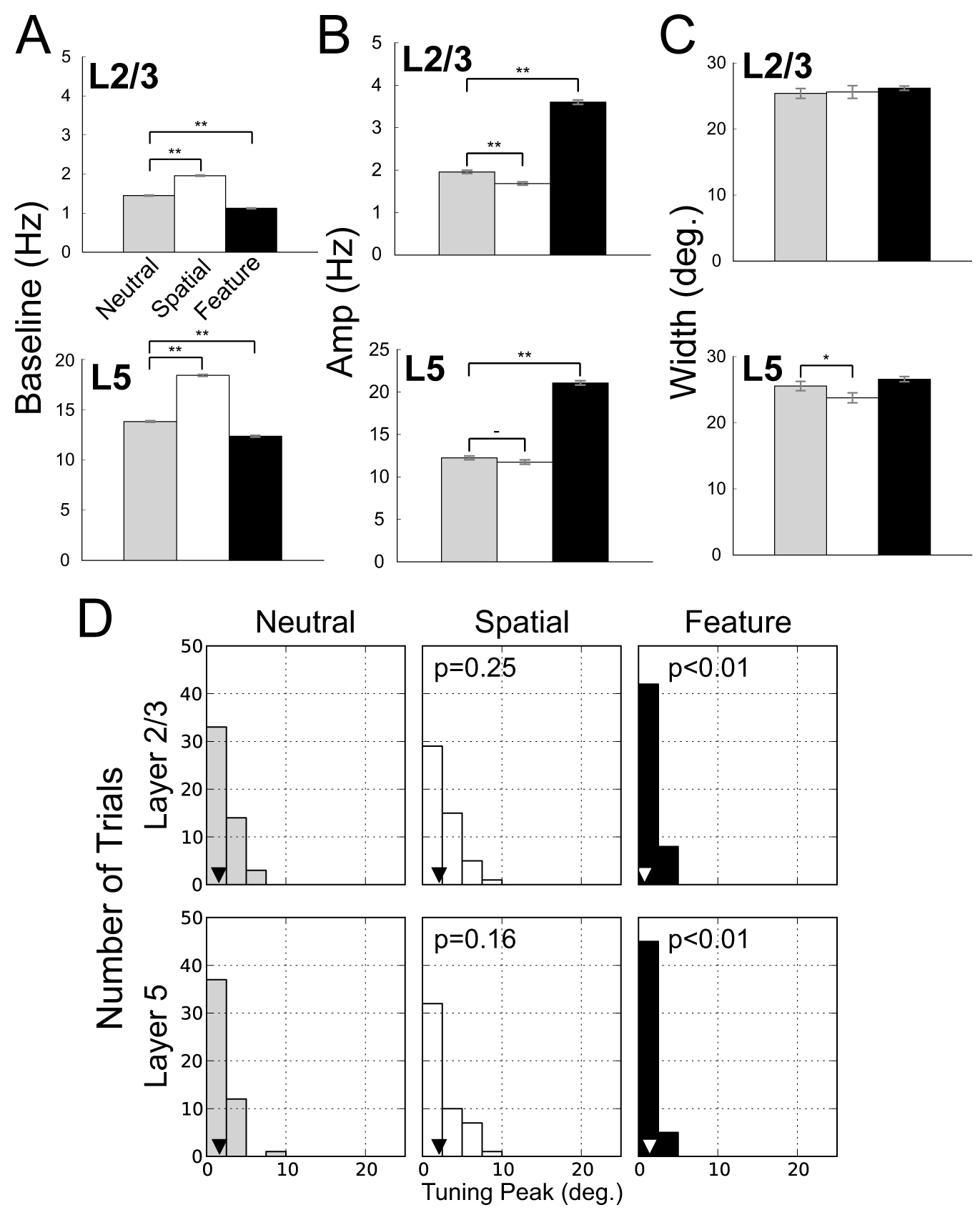

Supplement: Figure S5 — Statistical analyses of the orientation tuning curves for the modified model. A, B, C, The baselines, amplitudes and widths of the Gaussian tuning curves are shown for the responses of excitatory neurons in L2/3 (upper) and L5 (lower). The values of the Gaussian fitting parameters were obtained from 50 simulation trials in the neutral condition (gray bars), spatial attention (empty bars) and feature-based attention (filled bars). Asterisks indicate that the parameter values are significantly different from those in the neutral condition (t-test: ** for p<0.01; * for p<0.05; – for p<0.1). D, The histograms of the peak locations of the tuning curves in L2/3 and L5 are shown for a vertical bar stimulus. Triangles are the medians. We calculated P values for Mann-Whitney test to compare the histograms between the neutral condition and the two attentional conditions. (TIF) [file pone.0080788.s005.tif]

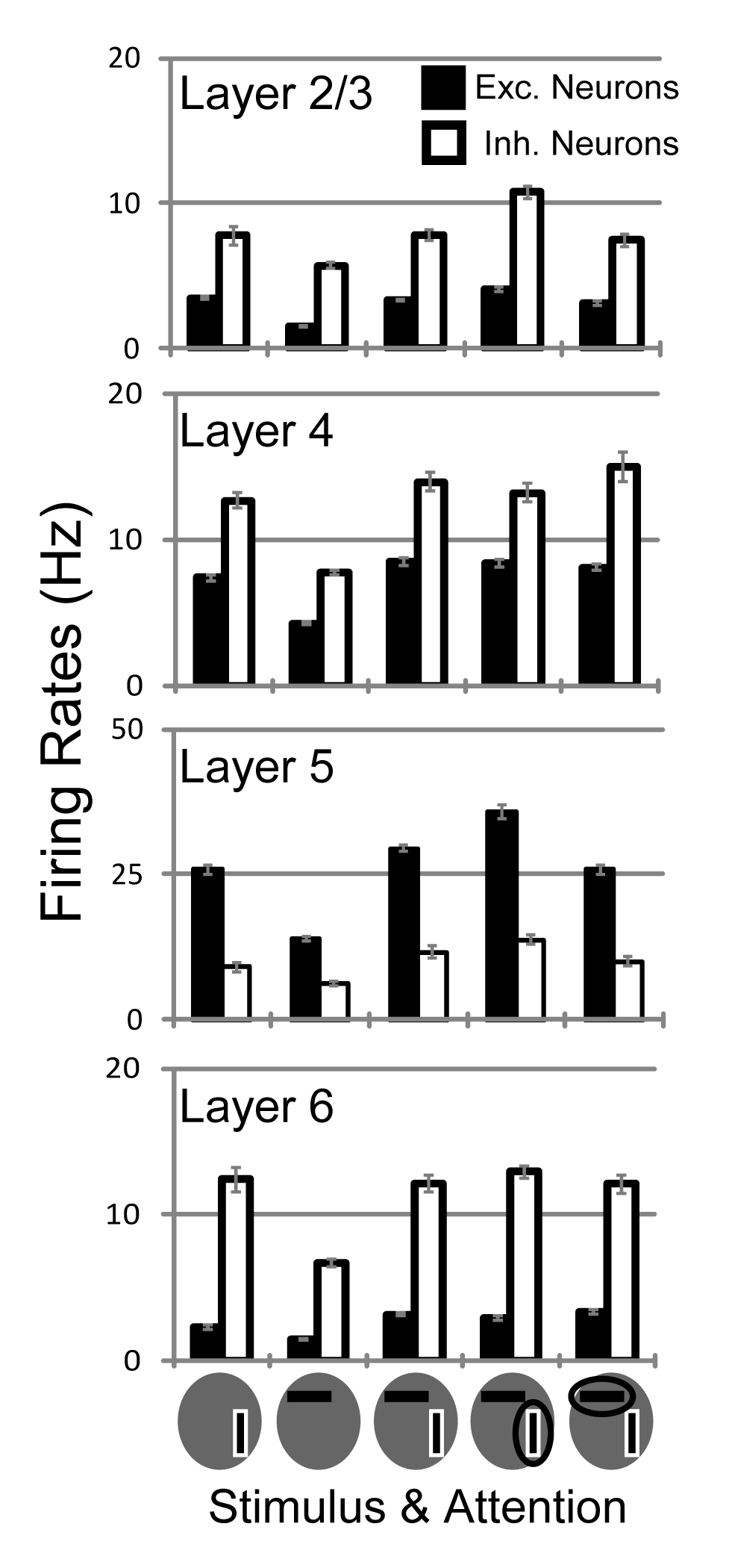

Supplement: Figure S6 — The averaged neuronal responses of the modified model for the biased competitions from 5 simulation trials. All the conventions are the same as those used in Figure S1. The modulation patterns in L2/3 and L5 are inconsistent with the results of physiological experiments [S13]. (TIF) [file pone.0080788.s006.tif]

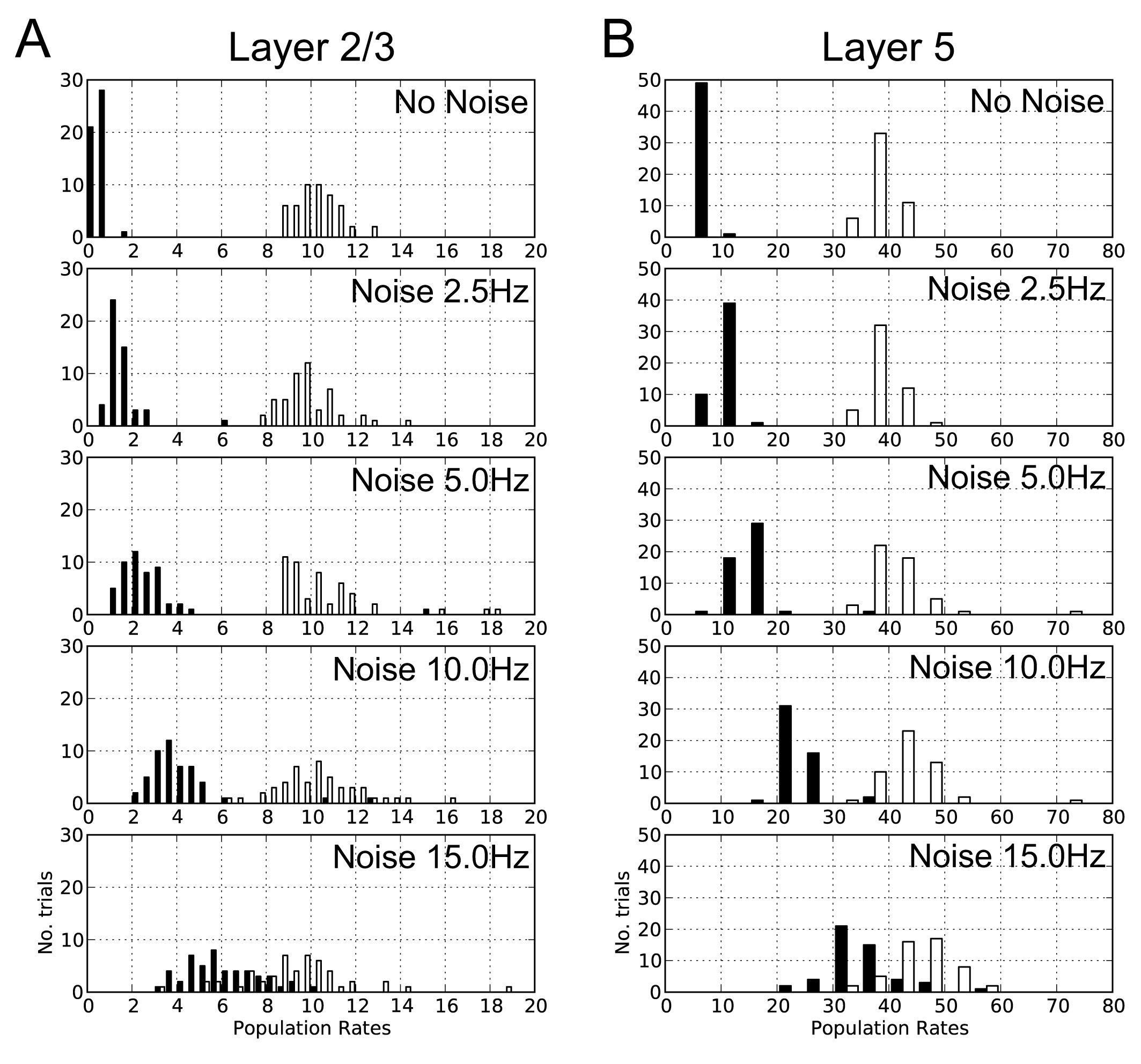

Supplement: Figure S7 — Frequency histograms of population firing rates on the neutral condition obtained from L2/3 A and L5 B at five different levels of external noise. The horizontal axis shows the amplitude of the population rates, and vertical axis indicates the number of trials. Each plot shows the levels of external noise. White bars depict population responses obtained from the preferred orientation, while black bars illustrate population responses to the non-preferred orientation. Each distribution is obtained from 50 simulation trials in the neutral condition. With increasing level of external noise, these two distributions were merged, which suggested the interferences of the accurate detection of a presented stimulus under the high level of noise. (TIF) [file pone.0080788.s007.tif]

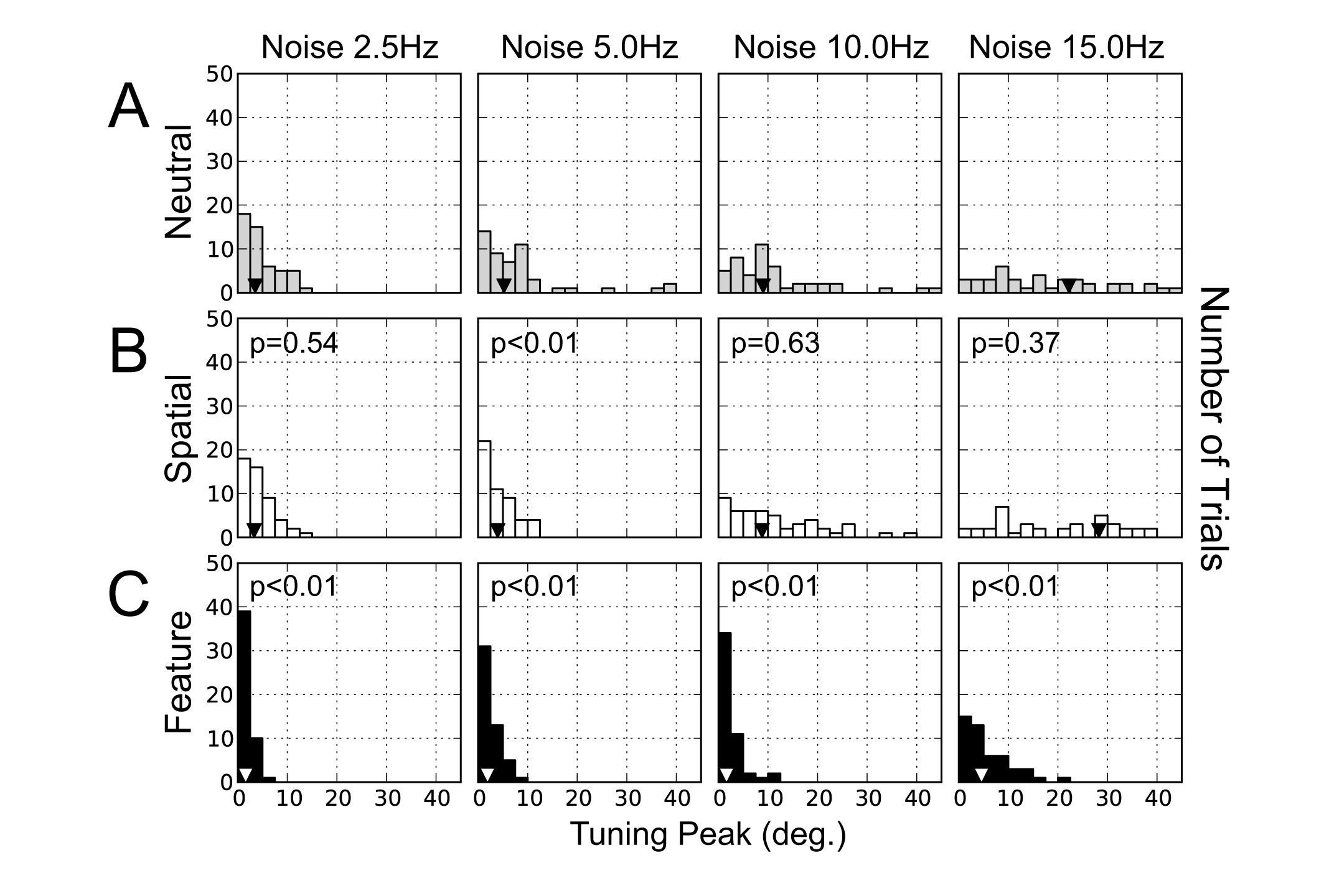

Supplement: Figure S8 — The histogram of the peak location of the tuning curves in L2/3 for the responses to the vertical bar with a variety of levels of external noise. Triangles are the medians. We calculated P values for Mann-Whitney test to compare the histograms between the neutral condition and the two attentional conditions. A , The frequency histogram of tuning peaks of neutral condition. With increasing levels of external noise, the frequency is widely distributed. B , The histogram of the peak location of spatial attention with respect to the vertical bar with various levels of external noise. C , The histogram of the peak location of feature-based attention. Regardless of the level of external noise, feature-based attention improves the detection of the presented orientation compared to neutral condition. (TIF) [file pone.0080788.s008.tif]

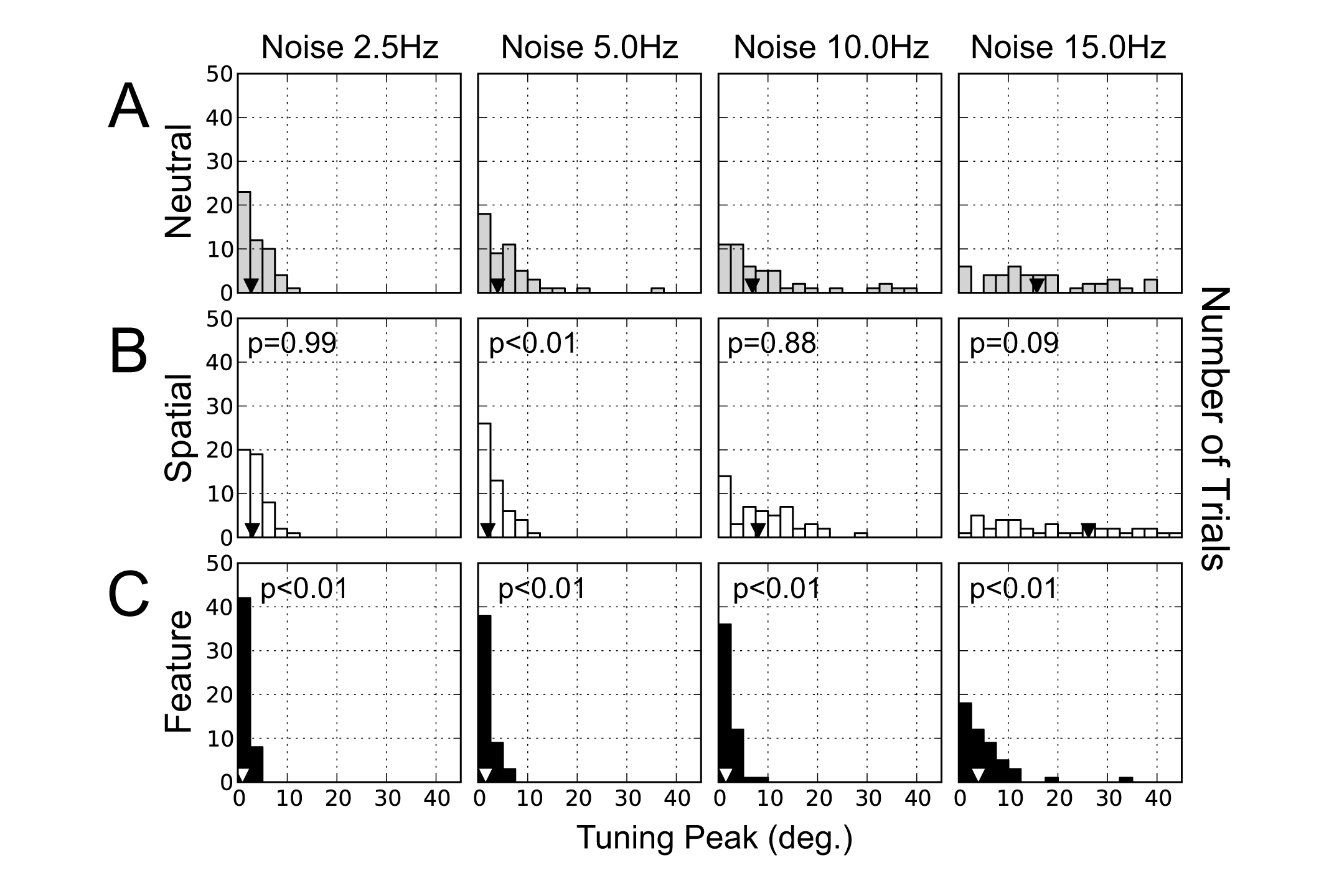

Supplement: Figure S9 — The histograms of the peak locations of the tuning curves in L5 obtained from same data sets shown in Figure S6. All the conventions are the same as those used in Figure S6. The results of statistical test (Mann-Whitney test) to compare between the neutral condition and two kinds of visual attention are identical to Figure S6. A, The frequency histogram of tuning peaks of neutral condition. B, The distribution of the peak locations for spatial attention. C, The histogram of the peak locations of feature-based attention. (TIF) [file pone.0080788.s009.tif]

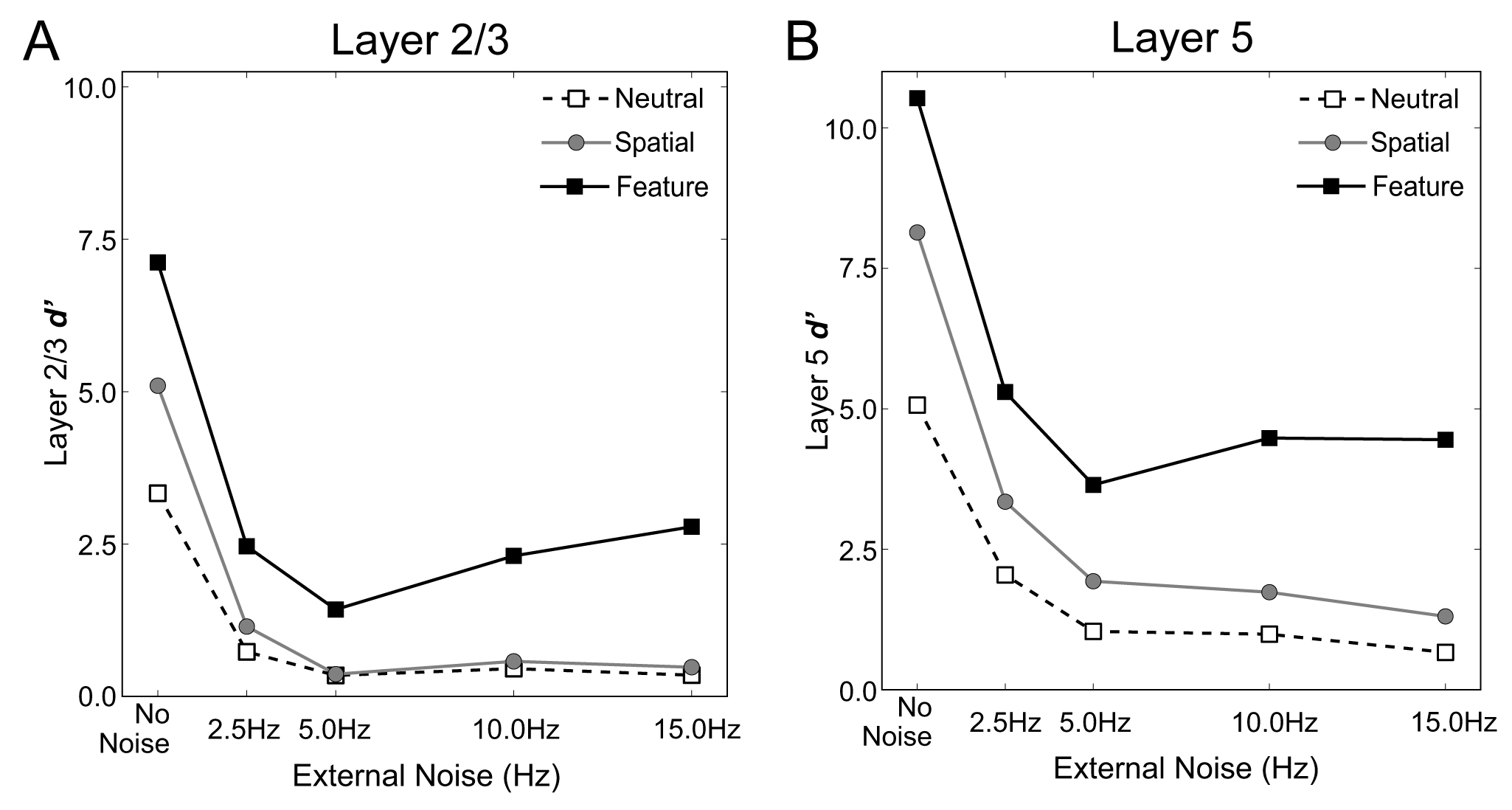

Supplement: Figure S10 — The performance of our model using the discriminability index ( d' ) for vertical and 22.5-degree oriented bars (fine discriminability). We computed the d' for L2/3 (A) and L5 (B). The dashed line shows the magnitude of d' in the neutral condition. Solid gray and black lines indicate the results of spatial and feature-based modes of attention, respectively. Effects of attention on discriminability show a similar tendency in L2/3 and L5. The decrement of this discriminability with increasing the levels of external noise was similar to the patterns of the discriminability between 2 orthogonal orientations. However, these magnitudes of d' between 2 similar oriented bars were markedly lower than that between orthogonal bars shown in Figure 7. (TIF) [file pone.0080788.s010.tif]
