# Supplementary material for: Spatial and Feature-Based Attention in a Layered Cortical Microcircuit Model
Source: PLoS One. 2013 Dec 6;8(12):e80788. doi: 10.1371/journal.pone.0080788 (PMC3855641; doi:10.1371/journal.pone.0080788)
Supplement: File S1 — This file contains Table S1-Table S9. Table S1, Model description after [S4] (Model Summary). Table S2, Model description after [S4] (Population). Table S3, Model description after [S4] (Connectivity). Table S4, Model description after [S4] (Neuron and synapse model). Table S5, Model description after [S4] (Input). Table S6, Model description after [S4] (Measurements). Table S7, Spike rates of excitatory background inputs. Table S8, Neuronal and synaptic model parameters (Connectivity). Table S9, Neuronal and synaptic model parameters (Neuron and Synaptic model). (DOCX) [file pone.0080788.s011.docx]

**Supplemental Tables**

**Table S1.**

| **A: Model Summary** | |
| --- | --- |
| **Populations** | nineteen; sixteen cortical populations and three input populations |
| **Topology** | --- |
| **Connectivity** | random connections |
| **Neuron model** | cortex: leaky integrate and fire, fixed voltage threshold, fixed absolute refractory period (voltage clamp), input: fixed rate Poisson populations |
| **Synapse model** | exponential-shaped postsynaptic currents |
| **Plasticity** | --- |
| **Input** | cortex: independent fixed-rate Poisson spike trains to all neurons |
| **Measurements** | spiking activity |

**Table S2.**

| **B: Popultions** | | |
| --- | --- | --- |
| **Type** | **Elements** | **Number of population** |
| Cortical network | iaf neurons | sixteen, eight per unit microcircuit, two per layer |
| Sensory input | Poisson population | two, one per unit microcircuit |
| Attentional input | Poisson population | one |

**Table S3.**

| **C: Connectivity** | |
| --- | --- |
| **Type** | random connections with independently chosen pre- and postsynaptic neurons; see Tables 1-4 for connection probabilities |
| **Weights** | fixed, drawn from Gaussian distribution |
| **Delays** | fixed, drawn from Gaussian distribution, multiples of sim. stepsize |

**Table S4.**

| **D: Neuron and Synapse Model** | |
| --- | --- |
| **Name** | iaf neuron |
| **Type** | leaky integrate-and-fire, exponential shaped synaptic current inputs |
| **Subthreshold dynamics** | $\tau_{m}\frac{dV}{dt}=-V+\frac{I(t)}{C_{m}}$ if $(t>t^{*}+\tau_{ref})$ $V(t)=V_{reset}$   else $I_{syn}(t)=we^{-t/\tau_{syn}}$  |
| **Spiking** | if $V(t-)<\theta\wedge V(t+)\geq\theta$  1. Set $t^{*}=t$, 2. Emit spike with time stamp $t^{*}$ |

**Table S5.**

| **E: Input** | | |
| --- | --- | --- |
| **Type** | **Target** | **Size** |
| Background input | iaf neurons | independent Poissonian spikes (see Table S7) |

**Table S6.**

| **F: Measurements** |
| --- |
| spiking activity of a subset of iaf neurons |

**Table S7.**

| **C: Inputs** | | | | |
| --- | --- | --- | --- | --- |
| **Background Input** | Layer 2/3 | Layer 4 | Layer 5 | Layer 6 |
| Exc. Neurons (# of fibers) | 2200 | 2200 | 2200 | 2200 |
| Inh. Neurons (# of fibers) | 1600 | 1600 | 1600 | 1600 |
| Background rate | 8 Hz | | | |

**Table S8.**

| **A: Connectivity** | |
| --- | --- |
| **Parameter** | **Value** |
| Excitatory synaptic weights *w*  (mean, standard deviation) | 175.6 pA, 17.6 pA |
| Inhibitory synaptic weights *-gw* (mean, standard deviation) | -702.5 pA, -70.3 pA |
| Excitatory synaptic delays (mean, standard deviation) | 1.5 ms, 0.75 ms |
| Inhibitory synaptic delays (mean, standard deviation) | 0.75 ms, 0.375 ms |

**Table S9.**

| **B: Neuron and Synapse Model** | |
| --- | --- |
| **Parameter** | **Value** |
| Membrane time constant $\tau_{m}$ | 10 ms |
| Postsynaptic current time constant $\tau_{syn}$ | 0.5 ms |
| Absolute refractory period $\tau_{ref}$ | 2 ms |
| Membrane capacity $C_{m}$ | 250 pF |
| Reset potential $V_{reset}$ | -65 mV |
| Fixed firing threshold $\theta$ | -50 mV |
