# Supplementary material for: Spatial and Feature-Based Attention in a Layered Cortical Microcircuit Model
Source: PLoS One. 2013 Dec 6;8(12):e80788. doi: 10.1371/journal.pone.0080788 (PMC3855641; doi:10.1371/journal.pone.0080788)
Supplement: Text S1 — Supporting Materials and Methods. (DOCX) [file pone.0080788.s012.docx]

**Supplementary Material for details of the neuronal models**

*Outline of the microcircuit model*

A single multi-layered cortical microcircuit of the cortical network model (Figure 2 in main text) consists of an excitatory and an inhibitory population in layers 2/3, 4, 5 and 6. The populations consist of current-based integrate-and-fire neurons with exponential synaptic currents, and the neurons are randomly connected. The connection probabilities correspond to a connectivity map that integrates a major part of the current knowledge on the cortical microcircuitry (see below for details [S1, S2]). Every population receives Poissonian background spike trains in addition to the specific sensory and attentional inputs. Synaptic parameters are chosen such that the average shape (rise time and width) of an excitatory postsynaptic potential resembles the in vivo situation [S3]. We draw delays from a Gaussian distribution (forcing delays to be positive and multiples of the simulation stepsize). The comprehensive model description according to [S4] is given in Table S1-S6. The number of neurons is given in Table 5, the connection probabilities in Tables 1-4 and 6-7 and the Poissonian background firing rates in Table S7. Table S8 and S9 contain all neuronal and synaptic model parameters.

*Connectivity map*

The connectivity map of the layer-specific multi-layered cortical microcircuit is based on the integrated data set, which is primarily based on the data derived from anatomical reconstructions by [S5] and the electrophysiological hit rate estimates from [S6]. Furthermore, it includes data from further electrophysiological studies (see references in [S7]] as well as information from photostimulation studies [S8, S9] and electron microscopy [S10]. The derivation of the integrated connectivity map takes into account specific differences of the underlying experimental procedures, as explained below [S1].

The anatomical data [S5] provides the relative number of synapses participating in a connection and the total number of synapses, depending on pre- and postsynaptic type, of area 17. The product of these measures gives the absolute numbers of synapses *K* participating in individual connections. Following [S5], we use the layer- and type-specific absolute numbers of neurons *N* from [S11]. For the connection probabilities *C_a_* between populations we assume that the synapses are randomly distributed without preventing multiple contacts (multapses) between any neuron pair. With *N^pre(post)^* being the number of neurons in the presynaptic (postsynaptic) population:

$C_{a}=1-\left(1-\frac{1}{N^{pre}N^{post}}\right)^{K}$. (1)

The often used expression

$C_{a}=\frac{K}{N^{pre}N^{post}}$ (2)

corresponds to first-order Taylor series approximation (in *1/(N^pre^N^post^)*), and is valid for *K/*(*N^pre^N^post^*) <<1.

For the electrophysiological data [S6] we use the hit ratio *R* as a direct measure of connection probabilities between populations. When multiple experimental estimates are available for the same connection type, we calculate a weighted sum of the independently measured hit ratios:

$C_{p}=\sum_{i} R_{i}Q_{i}/\left(\sum_{j} Q_{j}\right)$, (3)

where *Q_i_* is the number of tested pairs in the *i*th experiment.

Experimental values of *C_a_* and *C_p_* exhibit inconsistent mean values due to the different underlying methods. Therefore, we consider how the actual connection probability may depend on the distance between pre- and postsynaptic neurons using a gaussian model:

$C(r)=C_{0}\exp\left(\frac{-r^{2}}{2\sigma^{2}}\right)$. (4)

With *r* being the lateral distance and the parameters *C_0_* and $\sigma$ give the peak connection probability (at zero lateral distance) and the lateral spread of connections, respectively. We may assume that the anatomical and electrophysiological data correspond to random samplings of connections within cylinders of different sampling radiuses (*r_a_* > *r_p_*):

(5)

Since $2\pi C_{0}\sigma^{2}$ is the same in both equations, we obtain

, (6)

where $\tilde{C}_{a}=\lim_{r_{a}\to\infty}C_{a}\pi r_{a}^{2}$ is the anatomical area-corrected connection probability and $\tilde{C}_{p}=C_{p}\pi r_{p}^{2}$ its physiological counterpart. For $r_{a}>>\sigma$, we further obtain

. (7)

The zero-distance connection probability is then given as

. (8)

Once *C*(*r*) is given, we may use a layered microcircuit model of radius *r_m_* with a laterally homogeneous connection profile. Thus, the constant connection probability *C_m_* of the model is determined by the following equation:

. (9)

We use the above expression of *C_m_* in deriving the values of connectivity listed in Tables 1-4.

Furthermore, we correct for shortcomings of the individual experimental connectivity maps by consistent modifications of the target specificity of connections. According to the results of [S6, S8-S10], the projections from L2/3e to L4, from L5e to L2/3, from L2/3e to L6 and from L6e to L4, respectively, show a preferential selection of inhibitory targets.

We quantify the specificity of connections by the target specificity

$T=\frac{C^{post=e}-C^{post=i}}{C^{post=e}+C^{post=i}}$ (10)

For the above listed projections, the target specificity is predefined and the connection probabilities have to be estimated constrained by the previous estimates potentially ignoring the specificity. For the anatomical data, these underlying measures are the numbers of synapses that participate in a projection. For the physiological data, it is the determined connection probability of one of the two connections forming the projection

(typically the second connection has not been quantified).

Modifying the connection probabilities while conserving the total number of synapses of a projection represents a redistribution of the synapses to the targets neurons. Thereto, we determine the fraction of synapses that target excitatory neurons Δ as a function of the requested target specificity and constrained by the total number of synapses and the sizes of the presynaptic and the two postsynaptic populations. Using eq. (2) we find, with $C^{post=e}=\Delta K/N^{pre}N^{post=e}$ and $C^{post=i}=(1-\Delta)K/N^{pre}N^{post=i}$,

$\Delta=\frac{N^{post=e}}{N^{post=i}}\frac{1+T}{1-T}\left(1+\frac{N^{post=e}}{N^{post=i}}\frac{1+T}{1-T}\right)$.

The modification of the physiological data is straightforward because in all cases considered here only one connection probability is experimentally given so that we can estimate the unknown value based on eq. (10):

$C^{post=i(e)}=\left(\frac{1-T}{1+T}\right)^{+(-)1}C^{post=e(i)}$.

The derived excitatory map is consistent with the map recently reported by [S12].

**References for Supporting Information**

S1. Potjans TC, Diesmann M (2012) The Cell-Type Specific Cortical Microcircuit: Relating Structure and Activity in a Full-Scale Spiking Network Model. Cereb Cortex doi:10.1093/cercor/bhs358

S2. Wagatsuma N, Potjans TC, Diesmann M, Fukai T (2011) Layer-dependent Attentional Processing by Top-down Signals in a Visual Cortical Microcircuit Model. Front in Comp Neurosci doi:10.3389/fncom.2011.00031.

S3. Fetz E, Toyama K, Smith W (1991) Synaptic interactions between cortical neurons. In: A. Peters, editor. Cerebral Cortex, Volume 9, Chapter 1, New York and London: Plenum Press. pp. 1-47.

S4. Nordlie E, Gewaltig MO, Plesser HE (2009) Towards reproducible descriptions of neuronal network models. PLoS Comp Bio, 5, e1000456. doi:10.1371/journal.pcbi.1000456.

S5. Binzegger T, Douglas RJ, Martin KAC (2004) A quantitative map of the circuit of cat primary visual cortex. J Neurosci 24: 8441-8453.

S6. Thomson AM, West DC, Wang Y, Bannister AP (2002) Synaptic connections and small circuits involving excitatory and inhibitory neurons in layers 2-5 of adult rat and cat neocortex: triple intracellular recordings and biocytin labelling in vitro. Cereb Cortex 12: 936-953.

S7. Thomson AM, Lamy C (2007) Functional maps of neocortical local circuitry. Front in Neurosci doi: 10.3389/neuro.01.1.1.002.2007

S8. Dantzker JL, Callaway EM (2000) Laminar sources of synaptic input to cortical inhibitory interneurons and pyramidal neurons. Nat Neurosci 3: 701-707.

S9. Zarrinpar A, Callaway EM (2006) Local connections to specific types of layer 6 neurons in the rat visual cortex. J Neurophysiol 95: 1751-1761.

S10. McGuire BA, Hornung JP, Gilbert CD, Wiesel TN (1984) Patterns of synaptic input to layer 4 of cat striate cortex. J Neurosci 4: 3021-3033.

S11. Beaulieu C, Colonnier M (1983) The number of neurons in the different laminae of the binocular and monocular regions of area 17 in the cat. J Comparative Neurology 217: 337-344.

S12. Lefort S, Tomm C, Floyd Sarria JC, Petersen CC (2009) The excitatory neuronal network of the c2 barrel column in mouse primary somatosensory cortex. Neuron 61: 301-316.

S13. Reynolds JH, Chelazzi L, Desimone R (1999) Competitive mechanisms subserve attention in macaque areas V2 and V4. J Neurosci 19: 1736-1753.
